# Developing a dengue forecast model using machine learning: A case study in China

**DOI:** 10.1371/journal.pntd.0005973

**Published:** 2017-10-16

**Authors:** Pi Guo, Tao Liu, Qin Zhang, Li Wang, Jianpeng Xiao, Qingying Zhang, Ganfeng Luo, Zhihao Li, Jianfeng He, Yonghui Zhang, Wenjun Ma

**Affiliations:** 1 Department of Preventive Medicine, Shantou University Medical College, Shantou, China; 2 Guangdong Provincial Institute of Public Health, Guangdong Provincial Center for Disease Control and Prevention, Guangzhou, China; 3 Good Clinical Practice Office, Cancer Hospital of Shantou University Medical College, Shantou, China; 4 Guangdong Provincial Center for Disease Control and Prevention, Guangzhou, China; Institute for Disease Modeling, UNITED STATES

## Abstract

**Background:**

In China, dengue remains an important public health issue with expanded areas and increased incidence recently. Accurate and timely forecasts of dengue incidence in China are still lacking. We aimed to use the state-of-the-art machine learning algorithms to develop an accurate predictive model of dengue.

**Methodology/Principal findings:**

Weekly dengue cases, Baidu search queries and climate factors (mean temperature, relative humidity and rainfall) during 2011–2014 in Guangdong were gathered. A dengue search index was constructed for developing the predictive models in combination with climate factors. The observed year and week were also included in the models to control for the long-term trend and seasonality. Several machine learning algorithms, including the support vector regression (SVR) algorithm, step-down linear regression model, gradient boosted regression tree algorithm (GBM), negative binomial regression model (NBM), least absolute shrinkage and selection operator (LASSO) linear regression model and generalized additive model (GAM), were used as candidate models to predict dengue incidence. Performance and goodness of fit of the models were assessed using the root-mean-square error (RMSE) and R-squared measures. The residuals of the models were examined using the autocorrelation and partial autocorrelation function analyses to check the validity of the models. The models were further validated using dengue surveillance data from five other provinces. The epidemics during the last 12 weeks and the peak of the 2014 large outbreak were accurately forecasted by the SVR model selected by a cross-validation technique. Moreover, the SVR model had the consistently smallest prediction error rates for tracking the dynamics of dengue and forecasting the outbreaks in other areas in China.

**Conclusion and significance:**

The proposed SVR model achieved a superior performance in comparison with other forecasting techniques assessed in this study. The findings can help the government and community respond early to dengue epidemics.

## Author summary

Dengue epidemics have posed a great burden expanding of disease, with areas expanding and incidence increasing in China recently. It has remained challenging to develop a robust and accurate forecast model and enhance predictability of dengue incidence. Several state-of-the-art machine learning algorithms, including the support vector regression algorithm, step-down linear regression model, gradient boosted regression tree algorithm, negative binomial regression model, least absolute shrinkage and selection operator linear regression model and generalized additive model, were compared and evaluated to forecast dengue incidence in this study. The SVR model, based on selection by a cross-validation technique, was superior to other models assessed using weekly dengue surveillance data, Baidu search query data and meteorological data during 2011–2014 in Guangdong province. The high accuracy and robustness of the proposed SVR model to predict the occurrence of an outbreak was also validated using data from other provinces, including Yunnan, Guangxi, Hunan, Fujian and Zhejiang, spanning southern China. To the best of our knowledge, this is the first attempt to thoroughly evaluate different algorithms for dengue incidence prediction. Our identification of the optimal model will help to precisely track dengue dynamics in the country.

## Introduction

Dengue is a serious infectious disease and remains rampant across tropical and subtropical regions [1]. Primary dengue infection in humans often leads to a variety of clinical symptoms, from mild fever to potentially fatal dengue shock syndrome, and effective antiviral agents capable of treating dengue infection are not available at present [1]. *Aedes* mosquitoes, including *Aedes aegypti* and *Aedes albopictus*, serve as the main transmission vector of dengue viruses [2]. The impacts of variability in climate conditions such as temperature and precipitation on development rates and habitat availability for *Aedes aegypti* and *Aedes albopictus* larvae and pupae have been identified [3]. By affecting agent development and transmission vector dynamics, climate factors influences the spread of dengue. According to a recent analysis of the global distribution and burden of dengue virus, the number of dengue infections per year is estimated to be 390 million, of which nearly 96 million are symptomatic [4]. The estimated number of dengue infections has sharply increased over the past 50 years, resulting in a huge impact on human health around the world.

In China, dengue is a notifiable disease, and in recent years the area affected by dengue has expanded and the incidence has steadily increased [5]. According to the China Center for Disease Control and Prevention (CDC), the range of dengue incidence is from 0.0091 to 3.4581 per 100,000 people, with a total of 52,749 new cases of dengue having been reported during 2009–2014 [6]. In particular, a succession of dengue outbreaks occurred in several provinces including Guangdong, Yunnan, Fujian, and Guangxi during 2014 (S1 Fig) [6]. All of these provinces are located close to Southeast Asian countries including Laos, Vietnam, Thailand, Singapore and Malaysia, where dengue has been hyperendemic for decades and poses a large burden of disease [7–10]. However, dengue is still characterized as an imported disease in China due to localized transmission sparked by regular virus importations from returned travelers or visitors, rather than endemic transmission [5].

Guangdong, the most developed province located in southern China, experienced an unprecedented outbreak in 2014, and the number of cases reached the highest level over the past 25 years [5]. Our previous study showed that most of indigenous dengue cases occurred in the autumn of 2014, and the Pearl River Delta Region accounted for the majority of cases [11]. In addition to this remarkable spatial heterogeneity of cases, we observed a wide temporal variation of weekly dengue incidence ranging from 0 to 9,660 cases, which makes predicting dengue incidence difficult [11].

In the absence of an effective vaccine against dengue in China, accurate and early forecasts of dengue epidemics might allow for more effective targeting of control measures for the government. Since 2008, the China CDC has introduced the China Infectious Disease Automated-alert and Response System (CIDARS), which uses a time series moving percentile method based on historical data, for detecting dengue outbreaks in China [12]. This traditional method is overly dependent on the numbers of the routine surveillance data [12]. However, routine surveillance data is typically available with a 1- to 2-week lag [13]. Recently, several studies have explored the application of internet search terms to timely monitor disease outbreak and verify the usefulness and effectiveness of the approach [13–16]. The idea of applying internet search query data may contribute to enhancing predictability for dengue in Guangdong where dengue poses a great temporal cycling of incidence.

For dengue surveillance, several attempts have been made to develop robust predictive models for dengue incidence worldwide. Althouse et al. comprehensively assessed three regression models including step-down linear regression, gradient boosted regression tree model (GBM) and negative binomial regression model (NBM) for dengue incidence prediction in Singapore, and suggested the linear model selected by AIC step-down was superior to other models compared [16]. A more recent study achieved good performance by applying the least absolute shrinkage and selection operator (LASSO) algorithm to develop a real-time model to forecast dengue in Singapore [17]. In addition, generalized additive models (GAMs) were also used as valuable tools of risk assessment for dengue dynamics in previous studies [18, 19]. Furthermore, as a kind of the state-of-the-art and powerful machine learning algorithm, support vector regression (SVR) [20] displayed excellent performances in time series prediction. However, thorough comparisons of different predictive models and thus identifying an optimal model in China are still lacking. We aimed to construct an accurate forecast model to track the epidemic trajectory of dengue by comparing different prediction algorithms. This work addressed the gap by a) rigorously evaluating predictive performance of a variety of state-of-the-art algorithms using different assessment strategies and determining the optimal model, and b) combining dengue surveillance data, meteorological and internet query information with the proposed model for dengue incidence prediction in China.

## Materials and methods

### Data sources

#### Dengue case data

Weekly dengue case data of Guangdong, from 1 January 2011 to 31 December 2014, were obtained from the Guangdong Provincial CDC, which has access to the China National Notifiable Disease Surveillance System. Both of the imported and indigenous dengue cases were notified according to the surveillance system [21], and all of the cases during the study period were included in this study. Information recorded for each case includes basic demographic characteristics (gender, age, nationality and residential address), type of diagnosis (suspected, clinical, laboratory), virus serotype, and times of disease-related incidents (date of illness onset, diagnosis and death). All patient data were analyzed anonymously. Then the weekly number of dengue cases at the provincial level was calculated. In addition, the population census data of Guangdong in 2010, from the Statistics Bureau of Guangdong Province, was used to calculate dengue incidence. We further validated the models using dengue surveillance data from five other provinces comprised of Yunnan, Guangxi, Hunan, Fujian and Zhejiang (S1 Fig), which were at a high risk of dengue infection, in southern China during the study period. Monthly dengue surveillance data of the five provinces were at the provincial level and publicly available from the website (http://www.phsciencedata.cn/Share/index.jsp) of the Public Health Science Data Center managed by the China CDC. Clinical diagnosed and laboratory confirmed dengue cases were reported to the Chinese Ministry of Health and the data were aggregated and included in this analysis. All dengue cases were diagnosed according to the diagnostic criteria for dengue fever (WS216–2008) enacted by the Chinese Ministry of Health [22].

#### Meteorological data

Meteorological data for the areas during the study period were obtained from the China Meteorological Data Sharing Service System (http://cdc.nmic.cn/home.do). Weekly mean temperature (degrees Celsius), weekly average relative humidity and rainfall (millimeters) were extracted for the subsequent analyses. Meteorological data are recorded by monitoring stations widely distributed in China, and the weekly data of mean temperature, relative humidity and rainfall of a city were calculated using the area-weighted average method. Actually, these three meteorological factors were frequently used to develop time series forecast models for dengue and shown strong associations with dengue outbreaks in previous studies [23, 24].

#### Search query data

We obtained search query surveillance data from the Baidu Index website (https://index.baidu.com/) and constructed dengue search indexes (DSIs). Data were extracted on a weekly basis at a city and provincial level for the study period [13]. The search volume data of a term in a particular city is defined as the frequency of searching of a term keyed in by Baidu users in a city. Then the search volume data of a term in a province was summarized using the data from the cities.

### Statistical modeling

#### Keyword selection and search index construction

Previous studies proposed to chose the names or clinical symptoms of the studied diseases as the primary terms to search for more related keywords, which were usually obtained from a Chinese website (http://tool.chinaz.com/baidu/words.aspx) [15, 25]. Upon typing in 12 primary search terms, we obtained a group of 39 related keywords (S1 Table). We also established an auto-crawler software using Python to collect the search volume data of the keywords. The process of crawling search query data is depicted in S2 Fig. The Python scripts are available from the authors for academic usage.

Baidu search keywords used for this analysis were chosen by a sequence of selection procedures [13, 15]. Shi et al. establish a dengue forecast model using predictors with delayed effects in Singapore and verified its effectiveness [17]. According to the idea, we considered the predictors at lags of up to 8 weeks since this study covered a shorter period of time and the time lags were reasonable for our data. The process of constructing the DSI is given in S1 Text.

#### SVR and the compared models for dengue prediction

The SVR model has shown an excellent performance for time series prediction [26, 27]. We considered to use SVR for tracking dengue dynamics, and compared it with other time-series statistical models. This study implemented an *ε*-SVR approach, which uses a linear kernel function to predict a continuous dependent variable. For the SVR model, an optimal cost parameter *C* was selected to avoid overfitting and improve the predictive performance [20]. We performed a cross-validation approach with root-mean-square error (RMSE) as an indicator of model performance to select an optimal SVR model. Specifically, we trained several SVR models for different values of the *C* parameter, and chose the most superior one corresponding to the lowest RMSE value. This study tried values ranged from 0.005 to 1.0 with a span of 0.005 for the parameter *C* in the established SVR model.

For the step-down linear regression model, a backward elimination procedure was performed to search for an optimal subset of predictors that minimizes the Akaike information criterion (AIC) [28]. For the GBM, the number of trees was set to 1000 to ensure performance, and the rest of the parameters used the default values in the h2o package [29] of R. The GBM is a nonparametric algorithm with capability for regression by carving a high dimensional data space into mutually exclusive regions, and thus is robust in multicollinearity situations [30]. The NBM was chosen over Poisson regression due to over-dispersion of the search query data [16], and was fitted via penalized maximum likelihood method [31]. For the GAM, a natural cubic spline with 3 degrees of freedom was used for each predictor, and the model with the lowest generalized cross validation score was chosen [19]. The gam package was used to implement the GAM framework allowing for zero inflated Poisson data analysis. The LASSO algorithm is a shrinkage regression technique specially used to avoid the overfitting problem, and estimate the parameters of the model with low variability [32]. In this study, the 10-fold cross-validation approach [33] was performed to identify the optimal tuning parameter in the penalty function and then determined an optimal LASSO model.

Since there was a significant increase in cases of dengue in Guangdong during 2014, we first performed the models to predict the outbreak. In the compared models, the outcome variable was the weekly number of cases (natural log-transformed, with 1 added to avoid logging 0) [17]. We included the constructed DSIs, climate variables and their delayed effects as predictor variables in the model. The observed year and week as predictors were also included in the model to control for the long-term trend and seasonality, respectively. We further evaluated the performance of the models using dengue surveillance data from five other provinces. The same variables and parameter settings were used in the above-mentioned models.

#### Model comparison and validation

Candidate models were compared and validated using four scenarios. First, dengue surveillance data from the 1^st^ week of 2011 to the 41^st^ week of 2014 in Guangdong were used as training data to parameterize the models, and the last 12 weeks of the year to validate them. This strategy specifically evaluated the predictive accuracy of each model over a 12-week time horizon and compared their performance [17]. Second, in order to assess the performance of the models for forecasting the dengue outbreak in 2014, data from the 35^th^ week to 46^th^ week which covers the peak in dengue incidence were used to assess the prediction performance of the models. The models were evaluated using the RMSE [34], which is used to assess the differences between values predicted by a model and the actual values. If *y*_*t*_ is the actual number of dengue cases for time *t* when the prediction is made, and ${\hat{y}}_{t}$ is the number of cases predicted by a model, the RMSE for that model is $RMSE=\sqrt{\frac{\sum_{t=1}^{n}{\left({\hat{y}}_{t}-y_{t}\right)}^{2}}{n}}$, where *n* is the size of samples for prediction. A smaller RMSE indicates better predictive performance of a model. To briefly obtain prediction intervals from each model and compare their accuracy of covering the eventual data point, the estimated standard errors from step-down linear regression model were used to calculate the 95% prediction interval using the normal approximation method [35]. Then the goodness of fit of the models was assessed by means of the R-squared statistic [36], where the higher R-squared indicates the greater explanatory power in dengue incidence predicting. Moreover, to examine the adequacy of the models, we applied an autocorrelation function (ACF) and a partial autocorrelation function (PACF) to check if the residuals from the established models were independent and randomly distributed over time [37]. These analyses were performed for each of the twenty cities in Guangdong.

Third, in order to assess the ability of the models in tracking dengue dynamics, we applied an out-of-sample forecasting approach [17] to make 1-week-ahead predictions to achieve nearly real-time estimations of dengue incidence for the studied cities in Guangdong. The forecasts were characterized as an estimated incidence map of dengue, and compared with the true epidemic in the area.

Furthermore, the established models were validated using dengue surveillance data and internet search query from five other provinces at a high risk of dengue infection over the study period. The out-of-sample forecasting approach was employed and the 1-month-ahead predictions were obtained to compare their performance in tracking dengue dynamics in 2014. Predictive performance and goodness of fit of the models was also assessed using the RMSE and R-squared measurements, respectively. All statistical analyses were conducted in R version 3.0.2 (R Core Development Team).

## Results

Temporal characteristics of dengue cases, DSI, mean temperature, rainfall and relative humidity for each city in Guangdong province during 2011–2014 are presented in S3–S12 Figs. There was a sharp increase in dengue cases in the autumn of 2014 for each city. In particular, the Pearl River Delta cities had the most obvious increase in the number of the notified dengue cases in September and October, and most areas in Guangdong have hotter temperatures and more rain during the summer season. The fluctuating trend in DSI was fairly consistent with the epidemic activity of dengue.

**Fig 1:**
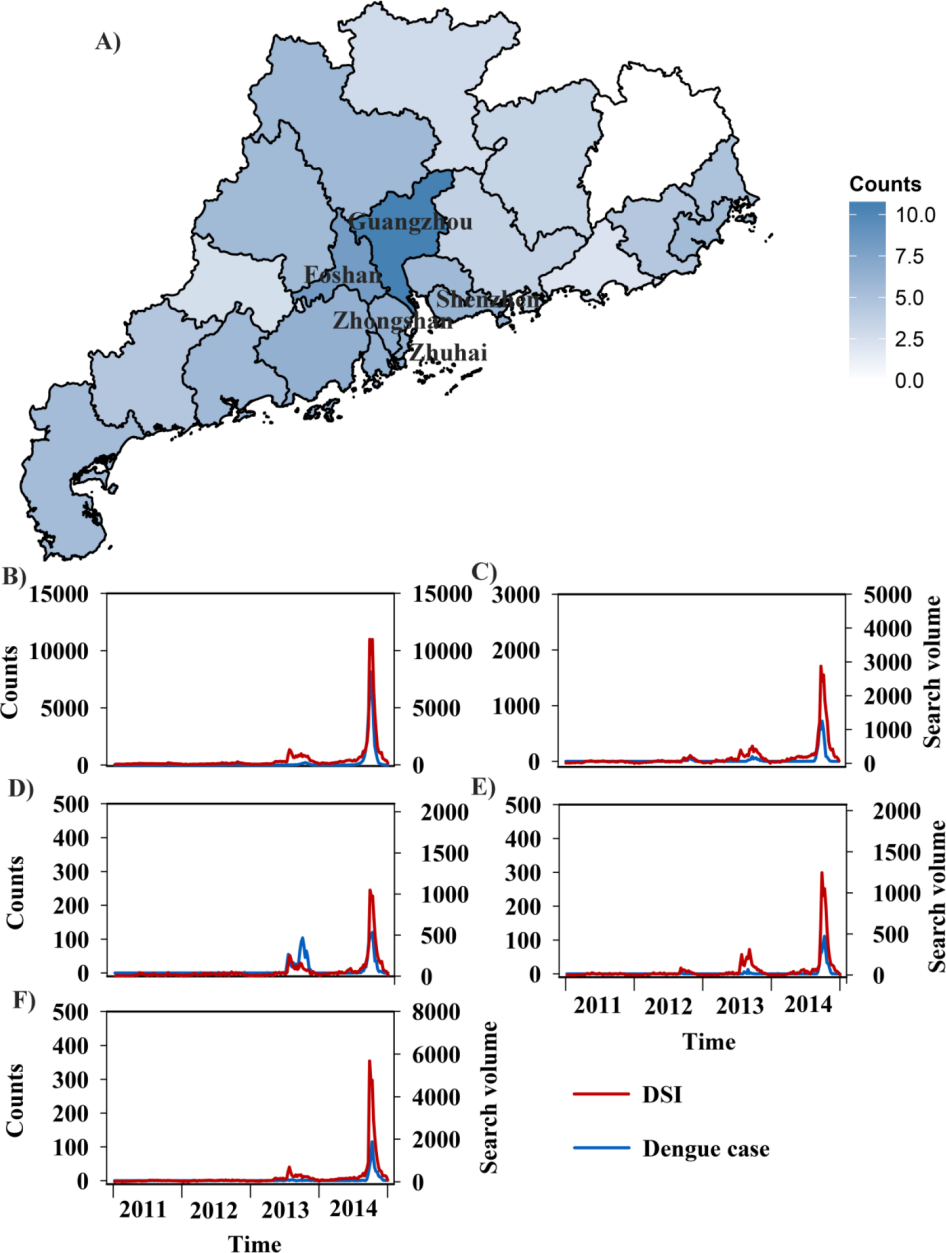
Spatiotemporal dynamics of dengue cases and dengue search index (DSI) during 2011–2014 in Guangdong province, China. (A) Geographical distribution of dengue cases in Guangdong province, China in 2014. (B) Time series of dengue cases and DSI in Guangzhou city. (C) Time series of dengue cases and DSI in Foshan city. (D) Time series of dengue cases and DSI in Zhongshan city. (E) Time series of dengue cases and DSI in Zhuhai city. (F) Time series of dengue cases and DSI in Shenzhen city.

In 2014, Guangdong accounted for about 96.3% of all notified dengue cases nationwide (S1 Fig). Spatiotemporal dynamics of dengue infections and DSIs during 2011–2014 in Guangdong is presented in Fig 1. Most of the dengue cases occurred in the Pearl River Delta region of Guangdong, especially for Guangzhou, Foshan, Zhongshan, Zhuhai and Shenzhen (Fig 1A). There was a close correlation between the number of dengue cases and the DSI in Guangdong (Fig 1 and S13 Fig).

**Fig 2:**
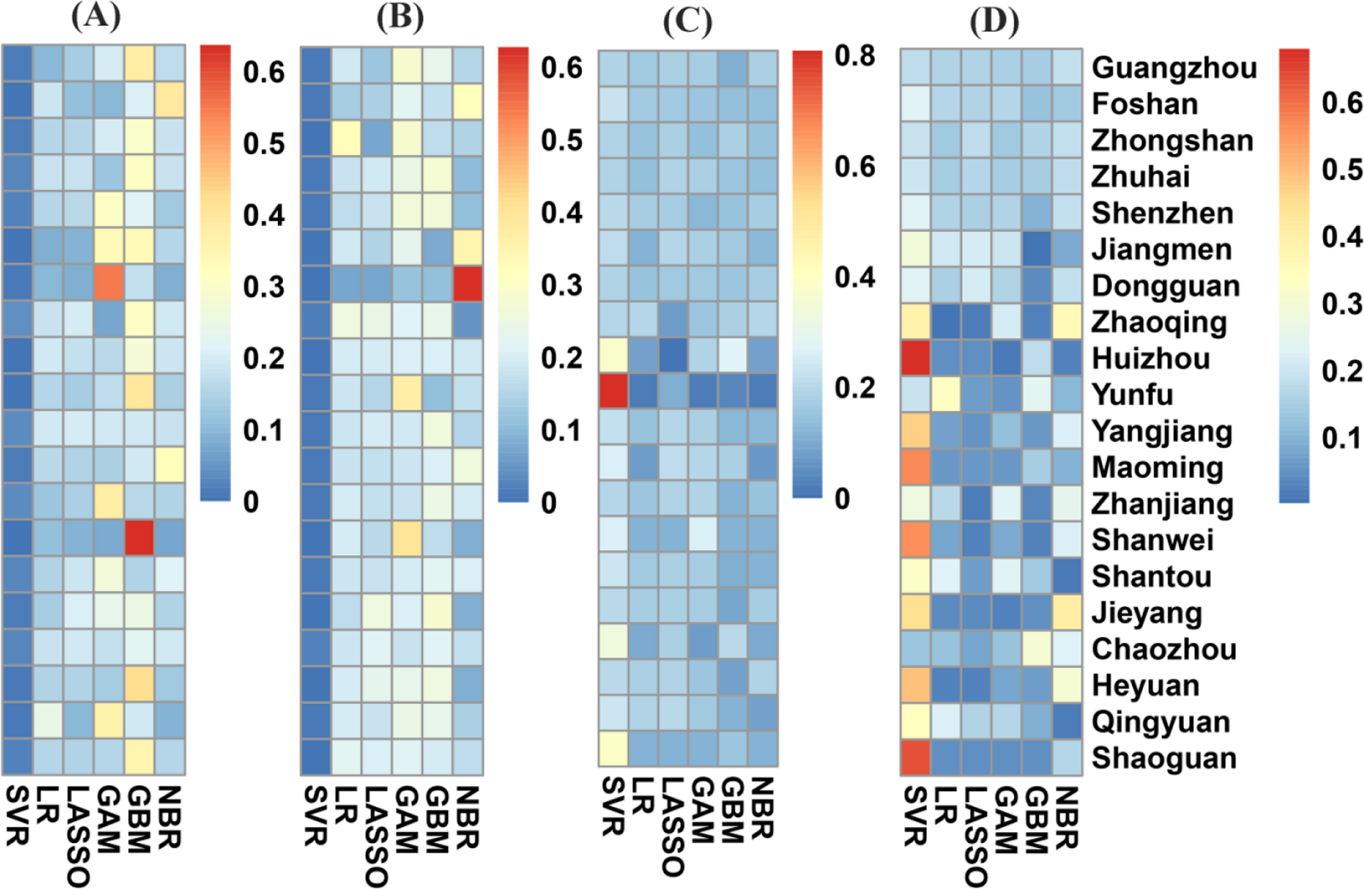
Comparison of prediction performance and goodness of fit of the models considered involving the support vector regression (SVR) model, step-down linear regression model, gradient boosted regression tree model (GBM), negative binomial regression model (NBM), least absolute shrinkage and selection operator (LASSO) linear regression algorithm and generalized additive model (GAM) using the root-mean-square error (RMSE) and R-squared statistic, respectively. (A) Data corresponding to the period between the 41^st^ to 53^rd^ weeks (the last 12 weeks) in 2014 was used to assess the models using the RMSE. (B) Data corresponding to the period between the 35^th^ to 46^th^ weeks which covers the outbreak in dengue incidence in 2014 was used to assess the models using the RMSE. (C) Data corresponding to the period between the 41^st^ to 53^rd^ weeks (the last 12 weeks) in 2014 was used to assess the models using the R-squared. (D) Data corresponding to the period between the 35^th^ to 46^th^ weeks which covers the outbreak in dengue incidence in 2014 was used to assess the models using the R-squared. The RMSE and R-squared values were standardized according to the specific city in Guangdong province.

**Fig 3:**
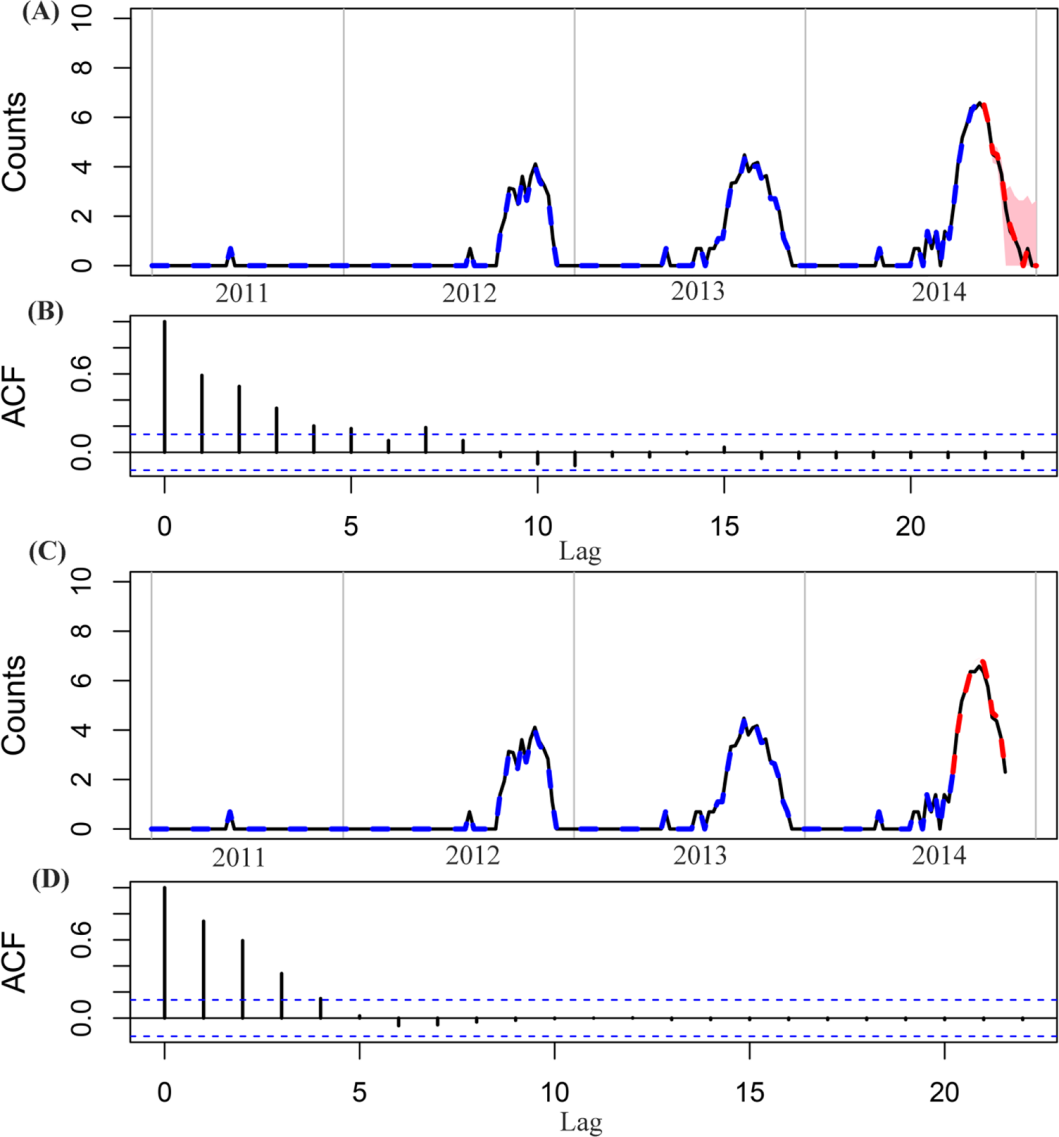
Observations and model predictions of dengue case counts in Foshan city, China, 2014. (A) Model forecasts using the SVR algorithm for the dengue epidemic period between the 41^st^ to 53^rd^ weeks (the last 12 weeks) in 2014. The black lines represent observed values, the blue dashed lines denote model-based fitted values, the red dashed lines correspond to model-based predicted values, and the pink contours represent the corresponding 95% prediction intervals. The observations and predictions of dengue case counts are expressed as a log-scale. (B) Residuals of the SVR model for the last 12 weeks forecasts were assessed using the autocorrelation function (ACF) plot. (C) Model forecasts using the SVR algorithm for the period between the 35^th^ to 46^th^ weeks which covers the outbreak in dengue incidence in 2014. (D) Residuals of the SVR model for the outbreak period forecasts were assessed using the ACF plot.

The relative predictive accuracy of dengue incidence and goodness-of-fit assessment for each model are shown in Table 1. The standardized RMSE and R-squared values for each city in Guangdong are shown in Fig 2. According to the model performance for the two prediction periods, the SVR model had the smallest RMSE values, irrespective of city. The results suggested that the SVR model outperformed other compared models and was chosen as the optimal model in this study. Results of goodness-of-fit suggested that the discrepancy between observed incidence and the incidence expected under the SVR model was smallest. Forecasts of the SVR model for the last 12 weeks and the outbreak period of dengue incidence in 2014, including 95% prediction intervals, for Foshan are presented in Fig 3. The epidemic during the last 12 weeks and the peak of the large 2014 outbreak were accurately forecasted by the SVR model. SVR model forecasts for the other four cities including Guangzhou, Zhongshan, Zhuhai and Shenzhen with a high risk of dengue infection are displayed in S14–S17 Figs, respectively. The ACF and PACF plots revealed that there was no autocorrelation in the residuals from the SVR approach established, and thus ensured the validity of the models (Fig 3 and S18 Fig). SVR algorithm consistently yielded the smallest prediction error rates for all the studied cities among the models compared, supporting the use of SVR to perform the forecasts. Additionally, the forecast accuracy of the SVR model increased as the value of parameter *C* got larger, and then quickly converged to a stable level, indicating the model had a good stability predictive ability (S19 Fig).

**Table 1:** Comparison of model performance and goodness-of-fit for support vector regression (SVR) model, step-down linear regression model (Linear), gradient boosted regression tree model (GBM), negative binomial regression model (NBM), least absolute shrinkage and selection operator (LASSO) linear regression algorithm and generalized additive model (GAM) by the means of root-mean-square error (RMSE) and R-squared, respectively. Two prediction periods were considered: 1) data corresponding to the period between the 41^st^ to 53^rd^ weeks (the last 12 weeks) in 2014 was used to validate the models; 2) data corresponding to the period between the 35^th^ to 46^th^ weeks which covers the outbreak in dengue incidence in 2014 was used to validate the models. Results are presented for five cities with a high risk of dengue infection, and the other cities in Guangdong province.

Measure	Prediction period	City	Model					
			SVR	Linear	LASSO	GAM	GBM	NBM
RMSE	The last 12 weeks	Guangzhou	16.2576*	109.9521	150.9228	218.0674	413.2917	182.2022
		Foshan	1.0483*	42.6509	25.9806	21.7453	47.6923	88.4364
		Zhongshan	0.3537*	3.7104	3.7638	4.7373	7.0461	4.3282
		Zhuhai	0.5717*	3.9115	3.9045	2.7538	6.7354	3.9376
		Shenzhen	0.8045*	6.1420	6.4693	12.0565	8.6777	5.0949
		Other cities studied	0.2681*	2.3806	2.0621	4.4973	3.4527	2.3305
	Outbreak period	Guangzhou	95.9668*	2204.7680	1378.6220	3215.8340	2691.7620	1764.1030
		Foshan	16.0143*	173.7577	181.8552	293.1263	223.1956	411.1545
		Zhongshan	1.1039*	89.4721	19.1110	78.5326	46.2534	39.9386
		Zhuhai	1.3978*	24.2412	25.9709	32.9678	38.1410	13.8852
		Shenzhen	1.6497*	26.8269	29.0679	43.6315	43.9624	18.4250
		Other cities studied	0.7876*	16.3118	14.9820	18.3629	15.3275	26.4680
R-squared	The last 12 weeks	Guangzhou	0.9990^§^	0.8513	0.9602	0.9315	0.5796	0.9411
		Foshan	0.9992^§^	0.7413	0.7142	0.7066	0.6054	0.6402
		Zhongshan	0.9948^§^	0.7932	0.9659	0.7416	0.9665	0.7704
		Zhuhai	0.9996^§^	0.7457	0.9699	0.9416	0.8287	0.7232
		Shenzhen	0.9983^§^	0.8307	0.8296	0.6099	0.7137	0.8423
		Other cities studied	0.9963^§^	0.5709	0.6463	0.6796	0.5620	0.5315
	Outbreak period	Guangzhou	0.9438^§^	0.8121	0.8170	0.8109	0.7736	0.9765
		Foshan	0.9441^§^	0.6670	0.6481	0.6794	0.5084	0.5748
		Zhongshan	0.9730^§^	0.6888	0.9039	0.6989	0.7929	0.9277
		Zhuhai	0.9804^§^	0.7074	0.8159	0.7329	0.7167	0.8946
		Shenzhen	0.9735^§^	0.6789	0.6278	0.6691	0.4033	0.7937
		Other cities studied	0.8865^§^	0.3081	0.2106	0.3361	0.1924	0.4224

* This indicates the values of RMSE of the SVR model were smallest.

^§^ This indicates the values of R-squared of the SVR model were largest.

**Fig 4:**
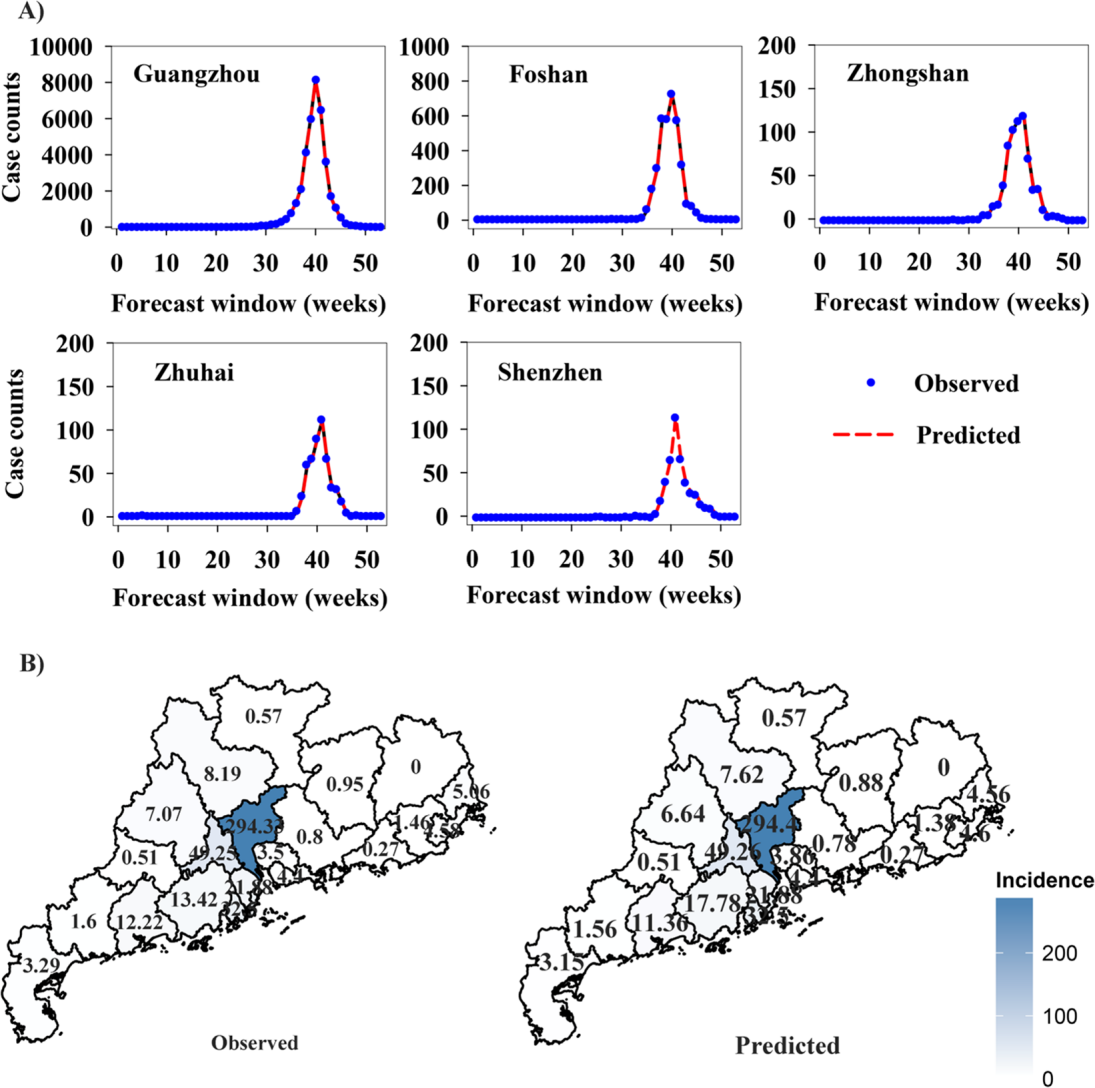
Observations and model predictions (1-week-ahead predictions) for the dengue outbreak in Guangdong, 2014. (A) Observations and model predictions of dengue case counts were only shown for five cities with a high risk of dengue infection in Guangdong province. In each panel, the blue points represent observed case counts, the red dashed lines denote model-based predicted values. Dynamic forecasts of dengue epidemics are presented in Video Files 1–5, respectively. (B) The actual dengue incidence map and that from the SVR model-based 1-week-ahead predictions in Guangdong, 2014. Incidence is expressed as the number of case counts per 100,000 people.

Predictions of dengue incidence in 2014 using an out-of-sample forecasting approach (1-week-ahead prediction for each forecast window) for the best fitted SVR model are shown in Fig 4. We observed an outstanding performance of the SVR model for detecting the peak of the large 2014 outbreak for the cities with a high risk of dengue infection (Fig 4A). Dynamic forecasts of dengue incidence for the five cities are presented in S1–S5 Videos. The estimated map of dengue incidence in 2014 for Guangdong province by the SVR model well described the truly epidemic proportions of this disease (Fig 4B). The ACF and PACF plots of the residuals from the fitted SVR models also revealed that there was no any autocorrelation in the residuals and the models had captured the patterns in the data quite well (S20 Fig).

**Fig 5:**
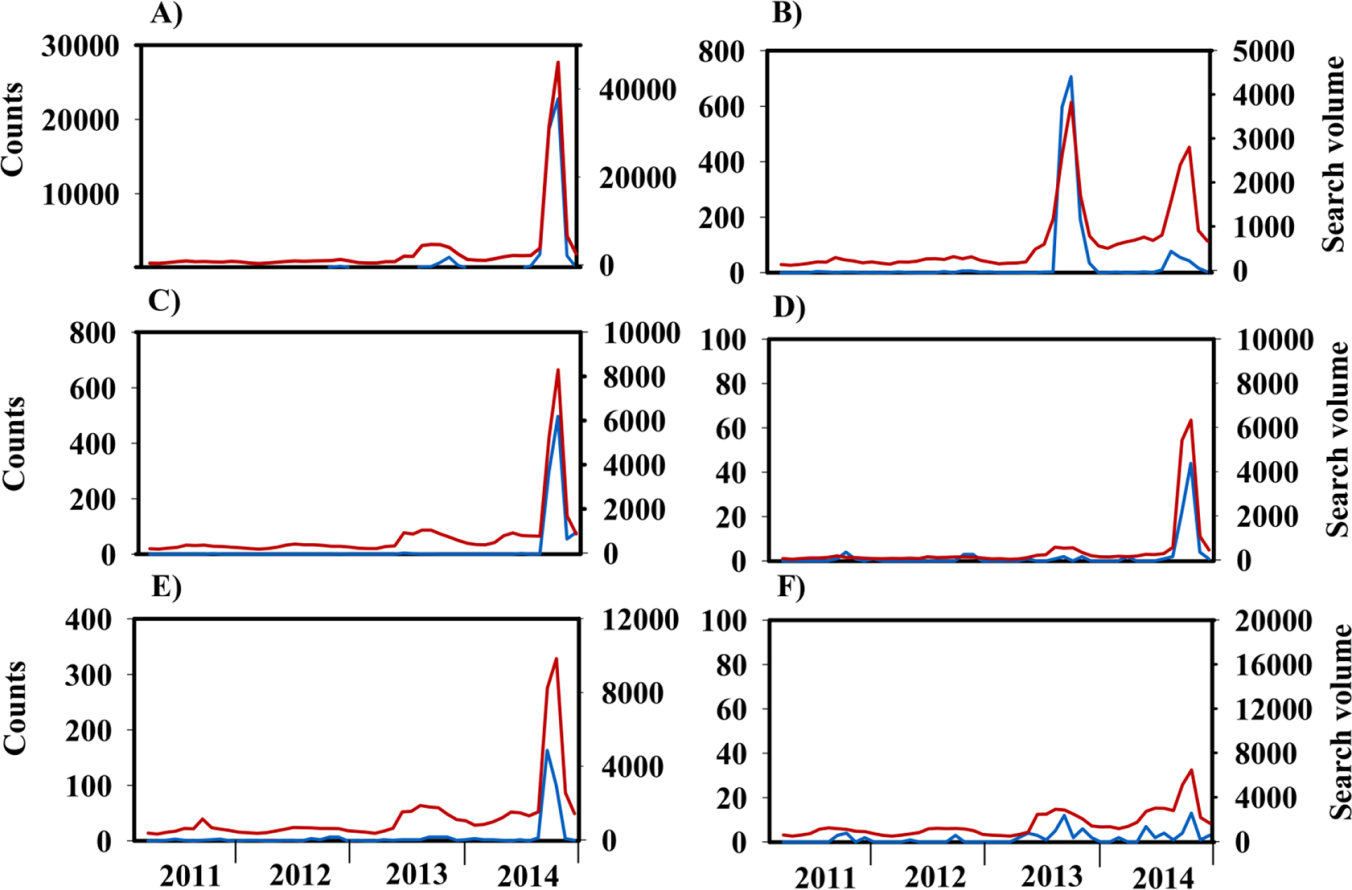
Temporal dynamics of dengue infection and dengue search index (DSI) during 2011–2014 in mainland China. (A) Time series of dengue cases and DSI in Guangdong province. (B) Time series of dengue cases and DSI in Yunnan province. (C) Time series of dengue cases and DSI in Guangxi province. (D) Time series of dengue cases and DSI in Hunan province. (E) Time series of dengue cases and DSI in Fujian province. (F) Time series of dengue cases and DSI in Zhejiang province. Blue lines represent time series of dengue case counts, and red lines represent time series of DSI, respectively.

**Fig 6:**
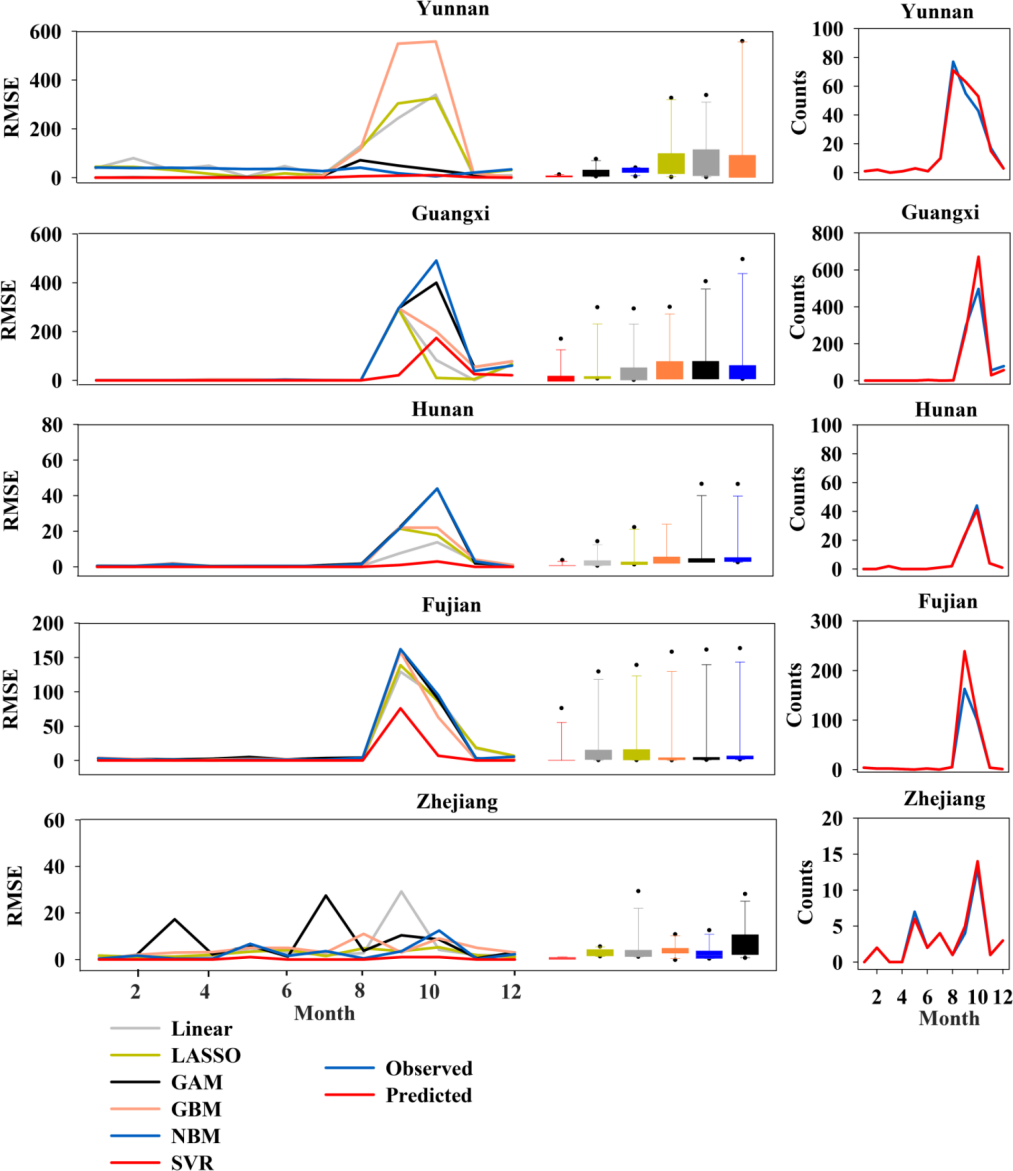
Comparison of prediction performance of the models including the support vector regression (SVR) model, step-down linear regression model, gradient boosted regression tree model (GBM), negative binomial regression model (NBM), least absolute shrinkage and selection operator (LASSO) linear regression algorithm and generalized additive model (GAM) by the means of root-mean-square error (RMSE). Left panel: comparison of 1-month-ahead predictions for the 2014 outbreak in Yunnan, Guangxi, Hunan, Fujian and Zhejiang which pose a high risk of dengue infection. In each panel, the RMSE values of different forecast windows for each model are summarized and presented as box plots. Right panel: the actual trend of 2014 dengue epidemics in Yunnan, Guangxi, Hunan, Fujian and Zhejiang are shown. In each panel, the blue lines represent observed case counts, and the red lines denote model-based predicted values.

To further validate the established models, we used dengue data from five other provinces, Yunnan, Guangxi, Hunan, Fujian and Zhejiang (S1 Fig), with a high risk of dengue infection in southern China. There was a high correlation between the epidemic activity of dengue infection and the trend in DSI in these areas (Fig 5A–5F). The assessment of predictions for single observations that were left out of the data set used to fit the model is presented in Fig 6. The results demonstrated a more competitive prediction by the SVR model relative to the other models, because the RMSE values of the SVR model were consistently smallest for the 1-month-ahead predictions in 2014, irrespective of the region investigated (Fig 6). The proposed SVR model had satisfactory prediction performance with large R-squared values for Yunnan (R-squared = 0.976), Guangxi (R-squared = 0.970), Hunan (R-squared = 0.997), Fujian (R-squared = 0.981) and Zhejiang (R-squared = 0.985) (Fig 6). It shows that the SVR model is a practical method to predict dengue dynamics in the five provinces.

## Discussion

This study demonstrates an efficient tool using a SVR algorithm to predict dengue outbreaks and track the epidemic trajectory in China. To the best of our knowledge, it is the first attempt to thoroughly evaluate the state-of-the-art algorithms for dengue prediction, and identify an optimal model that may help to complement the traditional surveillance for dengue dynamics.

Located in southern China, Guangdong has a subtropical humid monsoon climate and has frequent economic and cultural communication with the nations of Southeast Asia where dengue poses a great burden of disease. The climate, combined with Guangdong’s highly urbanized environment, favors the presence of *Aedes* mosquitoes and the transmission of dengue virus, thus making the area highly vulnerable to dengue outbreaks. In the absence of an effective vaccine against dengue in China, conducting a rapid survey on mosquito vector density and suppressing the vector population comprise the core of dengue-control programs at present [38]. Though a community-based integrated intervention strategy has been carried out to control dengue outbreaks in Guangdong [39], it is still important to enhance the predictability of dengue outbreaks that exhibit strong temporal cycling.

Although the China CDC has introduced the CIDARS for detection of dengue outbreaks, this method is overly dependent on numbers of notified dengue cases, and there is room to improve the predictive performance [12]. Moreover, due to an inherent defect in the routine surveillance approach, reports of the spread of dengue are delayed [13]. This may slow the quickly public health response to an impending outbreak of infectious disease to some degree. Taking these points into account, we believe that a statistical model holds the promise of being able to provide near real-time quantitative predictions of the occurrence and evolution of an outbreak of dengue, and may be used to efficiently guide the deployment of vector-control operations. Recent studies have exploited digital surveillance based on internet search behavior to timely monitor infectious diseases that have substantial seasonal and geographic variation [13–16]. Due to the increased availability and use of internet over the last decade, the behavior of people seeking information about health has been greatly changed by the availability of health-related information on the internet [40]. In China, according to the 39th Statistical Report on Internet Development, there are 73.1 million internet users in China until 2016, accounting for about 53.2% of the national population [41]. The remarkable increase in the internet use and search trends data of people is the basis for us being able to detect and track dengue outbreaks in the country.

However, evidence for a working statistical model that exhibits robust ability in the practice of dengue dynamics forecasting is still not available in China, especially for near real-time estimates of dengue epidemic activity in Guangdong, where the risk of dengue infections is high. Our study aimed to develop an accurate prediction tool for dengue outbreaks using machine learning in conjunction with internet search queries and meteorological data in China. Marcel et al. recently discussed the importance of internet-based disease surveillance for rapid disease outbreak detection, and proposed it as a powerful tool to complement traditional disease surveillance [42]. Our analysis found that specific search terms from Baidu are highly correlated with dengue incidence in China. Particularly, for Guangdong, the included search keywords showed a correlation of 0.91 with observed dengue incidence, which is basically consistent with previous studies [16].

We further demonstrate the feasibility of applying SVR in dengue incidence forecasting and show that the established SVR model is superior to the other models compared according to the results of the empirical analysis of this study. Our results, based on dengue surveillance data from five other high risk provinces of Yunnan, Guangxi, Hunan, Fujian and Zhejiang also demonstrate a more competitive performance by the SVR model. Our proposed method exhibited itself as a highly efficient tool to predict dengue incidence, and should have predictable positive impacts on the development of an early forecasting system for dengue outbreaks in China. Previous studies also show that a support vector machine-based model has high generalization performance and outperforms classical models in terms of prediction accuracy in Malaysia and Thailand, where the incidence of dengue outbreaks is also high [43, 44]. Our proposed SVR model further supports the support vector machine-based model as a highly efficient tool to predict dengue incidence.

The proposed SVR is a machine learning algorithm implementing the structural risk minimization inductive principle to minimize the generalized error bound and achieve good generalization in complex and noisy data [45]. In comparison to the considered models including step-down linear regression, GBM, NBM, LASSO and GAM, one of the main features of the SVR model is that it performs linear regression in the high-dimension feature space using *ε*-insensitive loss and tries to reduce model complexity, and handle different types of data sets with high prediction accuracy [46].

Although good generalization performance with SVR has been presented in this study when compared with other five models considered, this model can be abysmally slow in large-scale tasks since it has the extensive memory requirements [47]. Also, another important practical question of SVR lies in choice of the kernel [47]. Regarding the establishment of the SVR model herein, the most suitable kernel function for the dengue data should be considered. It has been suggested that linear kernel function is more robust to multicollinearity, and using the linear kernel function could achieve better performance than the RBF kernel function in case where the number of predictors is relatively large [48]. Additionally, the linear kernel has less complexity than other kernel functions because it has fewer hyperparameters and will be easier to understand. Therefore, the linear kernel function in SVR was used because it could effectively handle many variables in this analysis. Carefully tuning the cost parameter *C* for the established SVR model and selecting the most suitable value was also an important practical question to avoid overfitting and enhance predictive performance. In practice, the cost parameter *C* was varied through a wide range of values and the optimal performance assessed using cross-validation for verifying performance [49]. In this study, we applied a cross-validation technique to search the optimized value for the parameter *C*. By training several SVR models for different values of the parameter *C*, we chose the best model with the smallest RMSE.

Baidu is the most popular search engine in China, making it the most representative data source for tracking online behavior of Chinese people. However, several limitations related to internet search query based surveillance for infectious diseases should be mentioned. First, according to the 39th Statistical Report on Internet Development, the percentage of internet users in the rural areas has steadily increased and is responsible for 27.4% until 2016 [41]. Although the availability and popularity of the internet has grown greatly in the rural areas in recent years, the differences in the internet penetration between the rural and urban areas still exist and may influence the internet search queries based surveillance for dengue. Second, internet searching behavior is susceptible to the impact of media reports, which may affect the performance of the internet search term-based predictive model [50]. For example, due to a loss of resolution occurring as a result of media-driven interest that change search behavior, Google Flu Trends was reported to over-estimate the seasonal influenza [40]. In this study, we retrospectively assessed the performance of the proposed SVR model for dengue prediction. Prospective studies should be conducted to evaluate the impacts of media-driven interest or other events that change search behavior of people on the model in the future. In addition, although the variables of dengue case data, internet search surveillance data, meteorological data, and human population data were integrated and analyzed in this work, other sources of information on relevant indicators of risk, particularly evidence on mosquito density and herd immunity [16], may subsequently be incorporated in future studies. Furthermore, since annual population data in Guangdong province during the study period could not be obtained, the latest data of the 6^th^ population census in 2010 was used to calculate the observed and predicted dengue incidence. The variation of population during the study period might affect the estimates of dengue incidence in this study.

In conclusion, the present study demonstrates the utility of using SVR model to track dynamics of dengue outbreaks in China. The proposed SVR model achieves a superior performance in comparison with other forecasting techniques we assessed. The findings of this study will be useful for the government in identifying initiatives needed to strengthen dengue control.

## Supporting information

S1 Text Baidu search query data extraction, and search keyword selection and search index construction. (DOCX)

S1 Fig Five provinces at high risk of dengue infection in mainland China. (A) Geographic location of the provinces of Guangdong, Yunnan, Guangxi, Hunan, Fujian and Zhejiang. (B) Pie charts showing the percentage of the total number of dengue cases occurred in the country during the study period of 2011–2014 among the selected provinces.
(TIF)

S2 Fig A designed framework for crawling search keywords from the website of Baidu index using Python script in this study. (TIF)

S3 Fig Time series of weekly dengue cases, dengue search index (DSI), mean temperature, mean rainfall and mean relative humidity in the cities of Guangzhou and Foshan, Guangdong province during the study period of 2011–2014. (TIF)

S4 Fig Time series of weekly dengue cases, dengue search index (DSI), mean temperature, mean rainfall and mean relative humidity in the cities of Zhongshan and Jiangmen, Guangdong province during the study period of 2011–2014. (TIF)

S5 Fig Time series of weekly dengue cases, dengue search index (DSI), mean temperature, mean rainfall and mean relative humidity in the cities of Zhuhai and Shenzhen, Guangdong province during the study period of 2011–2014. (TIF)

S6 Fig Time series of weekly dengue cases, dengue search index (DSI), mean temperature, mean rainfall and mean relative humidity in the cities of Dongguan and Zhaoqing, Guangdong province during the study period of 2011–2014. (TIF)

S7 Fig Time series of weekly dengue cases, dengue search index (DSI), mean temperature, mean rainfall and mean relative humidity in the cities of Huizhou and Yunfu, Guangdong province during the study period of 2011–2014. (TIF)

S8 Fig Time series of weekly dengue cases, dengue search index (DSI), mean temperature, mean rainfall and mean relative humidity in the cities of Yangjiang and Maoming, Guangdong province during the study period of 2011–2014. (TIF)

S9 Fig Time series of weekly dengue cases, dengue search index (DSI), mean temperature, mean rainfall and mean relative humidity in the cities of Zhanjiang and Shanwei, Guangdong province during the study period of 2011–2014. (TIF)

S10 Fig Time series of weekly dengue cases, dengue search index (DSI), mean temperature, mean rainfall and mean relative humidity in the cities of Shantou and Jieyang, Guangdong province during the study period of 2011–2014. (TIF)

S11 Fig Time series of weekly dengue cases, dengue search index (DSI), mean temperature, mean rainfall and mean relative humidity in the cities of Chaozhou and Heyuan, Guangdong province during the study period of 2011–2014. (TIF)

S12 Fig Time series of weekly dengue cases, dengue search index (DSI), mean temperature, mean rainfall and mean relative humidity in the cities of Qingyuan and Shaoguan, Guangdong province during the study period of 2011–2014. (TIF)

S13 Fig Correlation between dengue cases notified and dengue search index (DSI) in 2014 in Guangdong, China. (A) Geographical distribution of dengue cases of 2014 in Guangdong. (B) Geographical distribution of DSI of 2014 in Guangdong. There was a significant correlation (Spearman correlation coefficient r = 0.91) between the geographical distribution of dengue incidence and that of DSI in Guangdong, China.
(TIF)

S14 Fig Observations and model predictions of dengue case counts in Guangzhou city, China, 2014. (A) Model forecasts using the SVR algorithm for the dengue epidemic period between the 41^st^ to 53^rd^ weeks (the last 12 weeks) in 2014. The black lines represent observed values, the blue dash lines denote model-based fitted values, the red dash lines correspond to model-based predicted values, and the pink contours represent the corresponding 95% prediction intervals. The observations and predictions of dengue case counts were expressed as a log-scale. (B) Residuals of the SVR model for the last 12 weeks forecasts were assessed using the autocorrelation function (ACF) plot. (C) Model forecasts using the SVR algorithm for the period between the 35^th^ to 46^th^ weeks which covers the outbreak in dengue incidence in 2014. (D) Residuals of the SVR model for the outbreak period forecasts were assessed using the ACF plot.
(TIF)

S15 Fig Observations and model predictions of dengue case counts in Zhongshan city, China, 2014. (A) Model forecasts using the SVR algorithm for the dengue epidemic period between the 41^st^ to 53^rd^ weeks (the last 12 weeks) in 2014. The black lines represent observed values, the blue dash lines denote model-based fitted values, the red dash lines correspond to model-based predicted values, and the pink contours represent the corresponding 95% prediction intervals. The observations and predictions of dengue case counts were expressed as a log-scale. (B) Residuals of the SVR model for the last 12 weeks forecasts were assessed using the autocorrelation function (ACF) plot. (C) Model forecasts using the SVR algorithm for the period between the 35^th^ to 46^th^ weeks which covers the outbreak in dengue incidence in 2014. (D) Residuals of the SVR model for the outbreak period forecasts were assessed using the ACF plot.
(TIF)

S16 Fig Observations and model predictions of dengue case counts in Zhuhai city, China, 2014. (A) Model forecasts using the SVR algorithm for the dengue epidemic period between the 41^st^ to 53^rd^ weeks (the last 12 weeks) in 2014. The black lines represent observed values, the blue dash lines denote model-based fitted values, the red dash lines correspond to model-based predicted values, and the pink contours represent the corresponding 95% prediction intervals. The observations and predictions of dengue case counts were expressed as a log-scale. (B) Residuals of the SVR model for the last 12 weeks forecasts were assessed using the autocorrelation function (ACF) plot. (C) Model forecasts using the SVR algorithm for the period between the 35^th^ to 46^th^ weeks which covers the outbreak in dengue incidence in 2014. (D) Residuals of the SVR model for the outbreak period forecasts were assessed using the ACF plot.
(TIF)

S17 Fig Observations and model predictions of dengue case counts in Shenzhen city, China, 2014. (A) Model forecasts using the SVR algorithm for the dengue epidemic period between the 41^st^ to 53^rd^ weeks (the last 12 weeks) in 2014. The black lines represent observed values, the blue dash lines denote model-based fitted values, the red dash lines correspond to model-based predicted values, and the pink contours represent the corresponding 95% prediction intervals. The observations and predictions of dengue case counts were expressed as a log-scale. (B) Residuals of the SVR model for the last 12 weeks forecasts were assessed using the autocorrelation function (ACF) plot. (C) Model forecasts using the SVR algorithm for the period between the 35^th^ to 46^th^ weeks which covers the outbreak in dengue incidence in 2014. (D) Residuals of the SVR model for the outbreak period forecasts were assessed using the ACF plot.
(TIF)

S18 Fig Residuals analysis using a partial autocorrelation function (PACF) for the established support vector regression (SVR) models. (A) PACF analysis for the SVR model forecasting the period between the 41^st^ to 53^rd^ weeks of 2014 in Guangzhou. (B) PACF analysis for the SVR model forecasting the period between the 35^th^ to 46^th^ weeks of 2014 in Guangzhou. (C) PACF analysis for the SVR model forecasting the period between the 41^st^ to 53^rd^ weeks of 2014 in Foshan. (D) PACF analysis for the SVR model forecasting the period between the 35^th^ to 46^th^ weeks of 2014 in Foshan. (E) PACF analysis for the SVR model forecasting the period between the 41^st^ to 53^rd^ weeks of 2014 in Zhongshan. (F) PACF analysis for the SVR model forecasting the period between the 35^th^ to 46^th^ weeks of 2014 in Zhongshan. (G) PACF analysis for the SVR model forecasting the period between the 41^st^ to 53^rd^ weeks of 2014 in Zhuhai. (H) PACF analysis for the SVR model forecasting the period between the 35^th^ to 46^th^ weeks of 2014 in Zhuhai. (I) PACF analysis for the SVR model forecasting the period between the 41^st^ to 53^rd^ weeks of 2014 in Shenzhen. (J) PACF analysis for the SVR model forecasting the period between the 35^th^ to 46^th^ weeks of 2014 in Shenzhen.
(TIF)

S19 Fig Tuning of the cost parameter C in the SVR model using cross-validation method. (A) Model forecasts for the dengue epidemic period between the 41^st^ to 53^rd^ weeks (the last 12 weeks) in 2014. (B) Model forecasts for the period between the 35^th^ to 46^th^ weeks which covers the outbreak in dengue incidence in 2014. A cross-validation approach with root-mean-square error (RMSE) as an indicator of model was performance to select an optimal SVR model. Several SVR models were trained for different values of the *C* parameter, and the most superior one corresponding to the lowest RMSE value was identified. Blue: Guangzhou. Red: Foshan. Orange: Zhongshan. Black: Zhuhai. Yellow: Shenzhen. The optimal values of RMSE for the SVR models are denoted using the pink solid dots.
(TIF)

S20 Fig Residuals analysis using an autocorrelation function (ACF) and a partial autocorrelation function (PACF) for the 1-week-ahead predictions of 2014 from support vector regression (SVR) models. (A) ACF analysis of the 1-week-ahead predictions in Guangzhou. (B) PACF analysis of the 1-week-ahead predictions in Guangzhou. (C) ACF analysis of the 1-week-ahead predictions in Foshan. (D) PACF analysis of the 1-week-ahead predictions in Foshan. (E) ACF analysis of the 1-week-ahead predictions in Zhongshan. (F) PACF analysis of the 1-week-ahead predictions in Zhongshan. (G) ACF analysis of the 1-week-ahead predictions in Zhuhai. (H) PACF analysis of the 1-week-ahead predictions in Zhuhai. (I) ACF analysis of the 1-week-ahead predictions in Shenzhen. (J) PACF analysis of the 1-week-ahead predictions in Shenzhen.
(TIF)

S1 Table Search keywords from Baidu index website used in this study. (DOCX)

S1 Video Dynamic 1-week-ahead forecasts of dengue incidence of 2014 in the city of Guangzhou, Guangdong. (MP4)

S2 Video Dynamic 1-week-ahead forecasts of dengue incidence of 2014 in the city of Foshan, Guangdong. (MP4)

S3 Video Dynamic 1-week-ahead forecasts of dengue incidence of 2014 in the city of Zhongshan, Guangdong. (MP4)

S4 Video Dynamic 1-week-ahead forecasts of dengue incidence of 2014 in the city of Zhuhai, Guangdong. (MP4)

S5 Video Dynamic 1-week-ahead forecasts of dengue incidence of 2014 in the city of Shenzhen, Guangdong. (MP4)

S1 Data Dengue data. (CSV)
