# Supplementary material for: Developing a dengue forecast model using machine learning: A case study in China
Source: PLoS Negl Trop Dis. 2017 Oct 16;11(10):e0005973. doi: 10.1371/journal.pntd.0005973 (PMC5658193; doi:10.1371/journal.pntd.0005973)
Supplement: S1 Text — (DOCX) [file pntd.0005973.s001.docx]

**S1 Text. Baidu search query data extraction, and search keyword selection and search index construction.**

Below we describe the details about Baidu search query data extraction, and search keyword selection and search index construction in this study.

Baidu is the most popular search engine in China, making it the most representative data source for tracking online behavior of Chinese people. We extracted search query surveillance data from the Baidu Index website (https://index.baidu.com/) and constructed dengue search indexes (DSIs). The website contains logs of online search query volume for numerous keywords searched by Baidu users. Search data were extracted on a weekly basis, at a city, and province level for the study period. For search queries-based surveillance, keyword selection is an important issue since it directly affects the predictive ability of the method. Previous studies generally chose the names or clinical symptoms of the studied diseases as the primary terms to search for more related keywords. We obtained the related keywords from a Chinese website (http://tool.chinaz.com/baidu/words.aspx). The recommended keywords were comprehensively abstracted from different sources including Baidu, portal websites and social blogs. Upon typing in 12 primary search terms respectively, we got 39 related keywords (Table S1). Then, an auto-crawler software using Python was written to collect daily search volumes of the keywords. The process of crawling search query data is depicted in Fig S2. The Python scripts can be available from the authors for academic usage.

Since some keywords may not be closely associated with dengue epidemics, we further filtered them by the following steps: (i) the selected keywords should represent factors that might affect the dengue occurrence; (ii) the search volume data for each keyword could be presented as a sequential time series with a specific resolution of time (e.g., daily, weekly or monthly), and those with a search volume of zero were eliminated; (iii) Spearman’s rank correlation coefficients (*ρ*) were used to assess the relationship between dengue epidemics and search trend for each keyword using different time lags. Frequently, predictors with delayed effects were simultaneously considered in prediction models, and this study allowed the effect of a single predictor to have lags of up to 8 units of time. Keywords with correlation coefficients less than 0.4 in each time lag and those correlations that were statistically insignificant (*P* > 0.05) were excluded.

The remaining keywords were used to construct a DSI for each time lag. The DSI was defined as follows:

$DSI_{l}=\sum_{i=l}^{n} weight_{li}*keyword_{li}$,

where $weight_{li}$ represents the weight of each keyword and is calculated using the formula of $weight_{li}=\frac{\rho_{li}}{\sum_{i=1}^{n} \rho_{li}}$, $keyword_{li}$ represents the *i*^th^ keyword, *l* denotes the time lag for each predictor, *n* is the number of keywords at each time lag, and *ρ* is the Spearman’s rank correlation coefficient to assess the correlation between dengue case counts and search volumes for each keyword.

**References**

1. Yuan Q, Nsoesie EO, Lv B, Peng G, Chunara R, Brownstein JS. Monitoring influenza epidemics in china with search query from baidu. *Plos One* 2013, **8**(5)**:** e64323.

2. Gu Y, Chen F, Liu T, Lv X, Shao Z, Lin H*, et al.* Early detection of an epidemic erythromelalgia outbreak using Baidu search data. *Scientific Reports* 2015, **5:** 12649.

3. Shi Y, Liu X, Kok SY, Rajarethinam J, Liang S, Yap G*, et al.* Three-Month Real-Time Dengue Forecast Models: An Early Warning System for Outbreak Alerts and Policy Decision Support in Singapore. *Environmental Health Perspectives* 2015, **124**(9)**:** 1369-1375.

4. Kang M, Zhong H, He J, Rutherford S, Yang F. Using Google Trends for influenza surveillance in South China. *Plos One* 2013, **8**(1)**:** e55205.
