# Supplementary figures and images for: Developing a dengue forecast model using machine learning: A case study in China
Source: PLoS Negl Trop Dis. 2017 Oct 16;11(10):e0005973. doi: 10.1371/journal.pntd.0005973 (PMC5658193; doi:10.1371/journal.pntd.0005973)

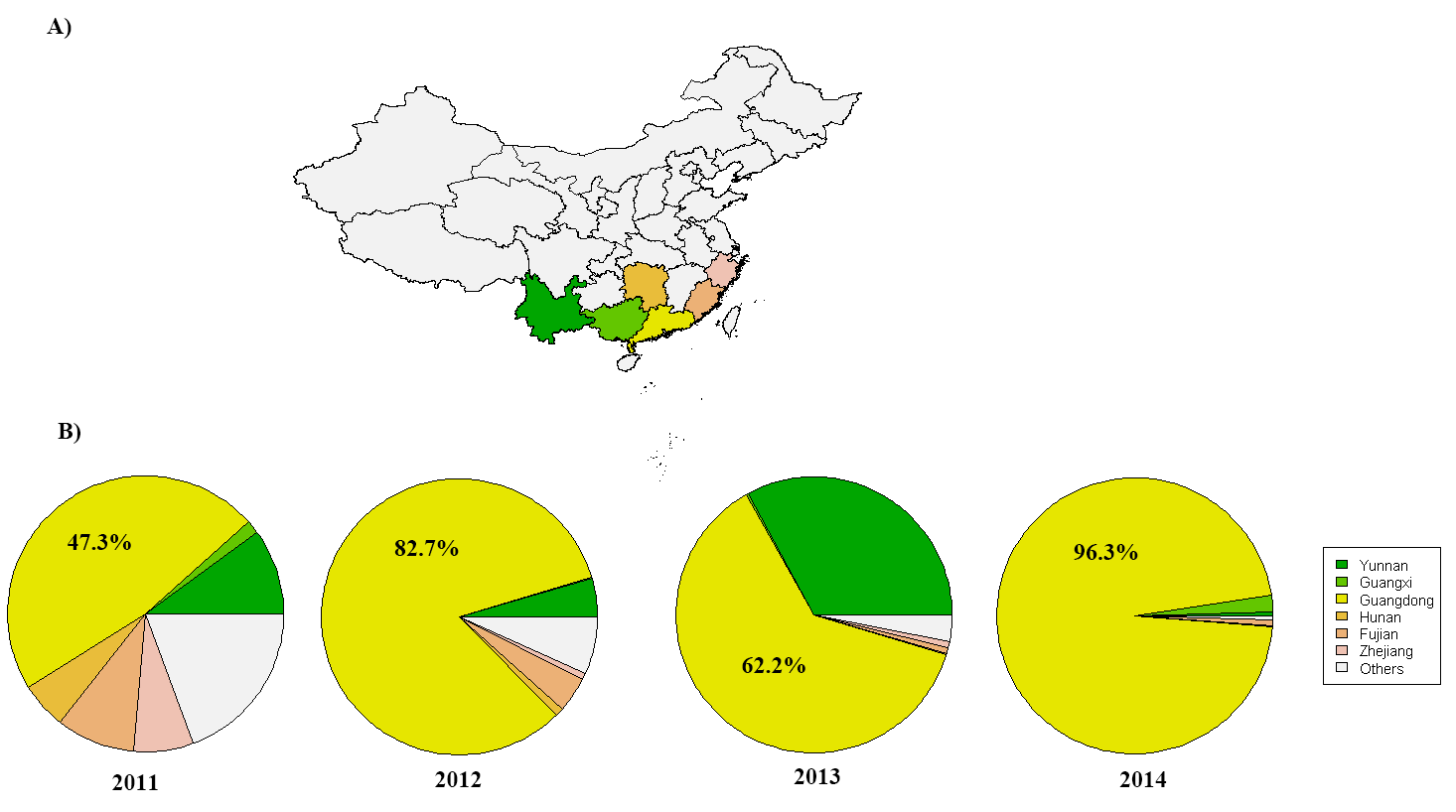

Supplement: S1 Fig — (A) Geographic location of the provinces of Guangdong, Yunnan, Guangxi, Hunan, Fujian and Zhejiang. (B) Pie charts showing the percentage of the total number of dengue cases occurred in the country during the study period of 2011–2014 among the selected provinces. (TIF) [file pntd.0005973.s002.tif]

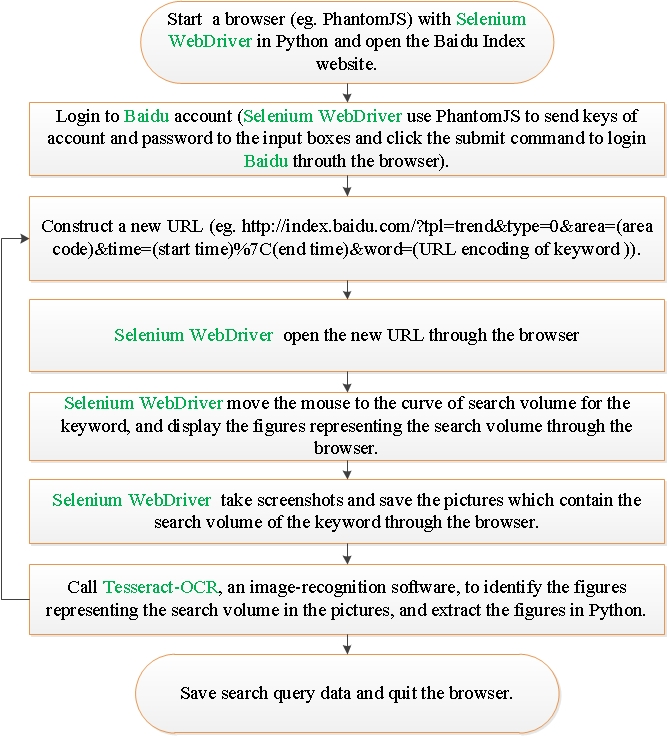

Supplement: S2 Fig — (TIF) [file pntd.0005973.s003.tif]

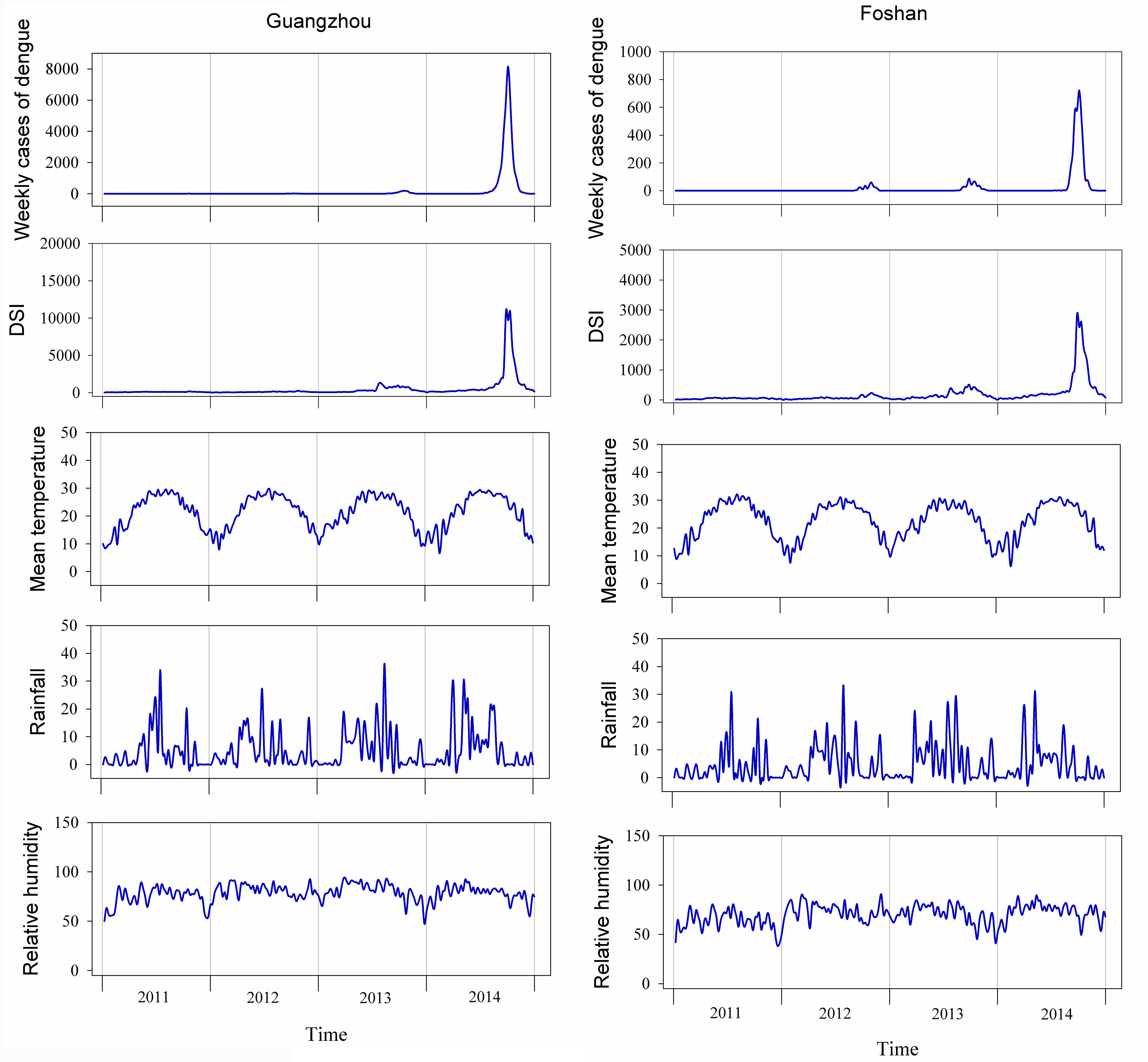

Supplement: S3 Fig — (TIF) [file pntd.0005973.s004.tif]

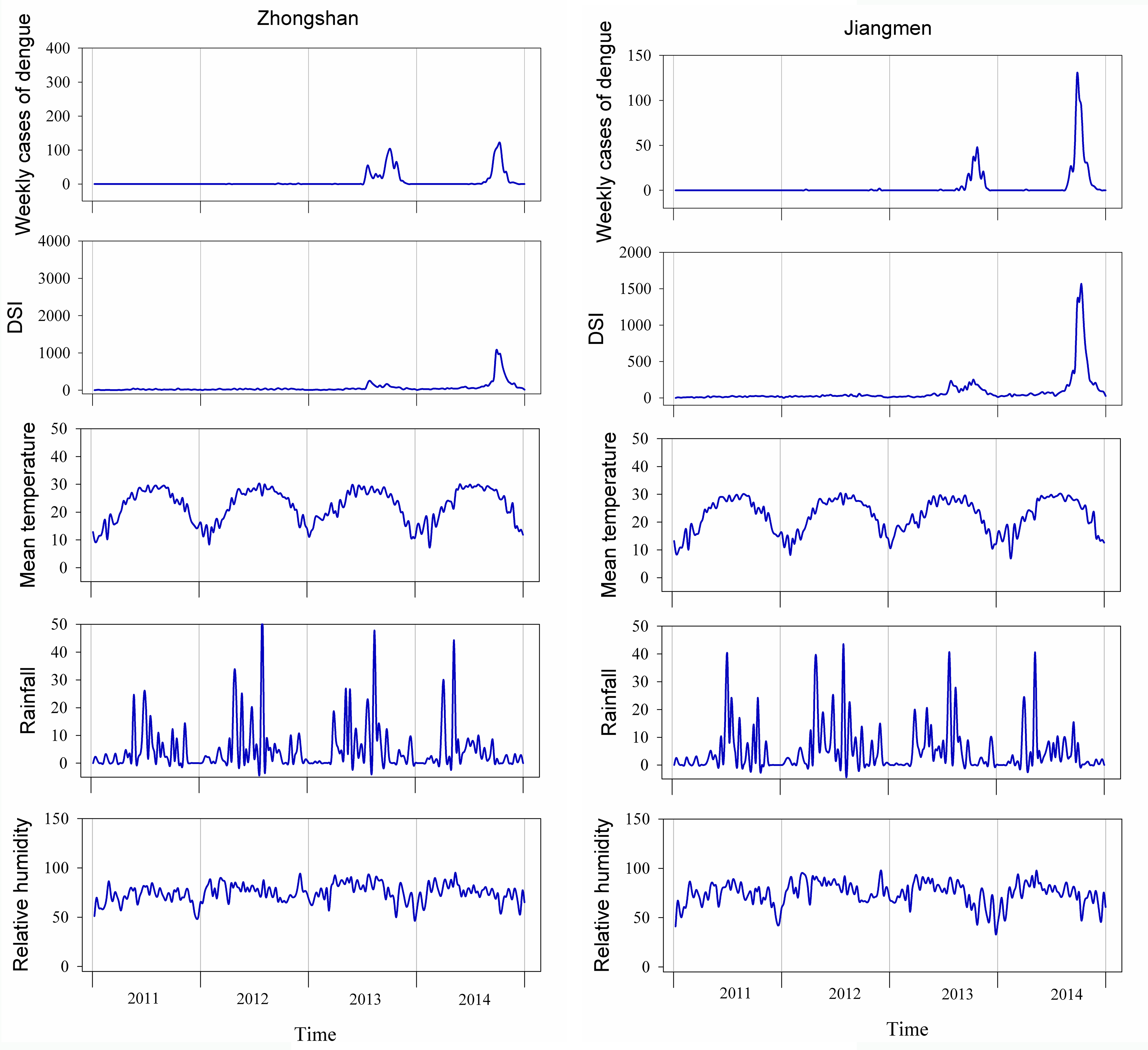

Supplement: S4 Fig — (TIF) [file pntd.0005973.s005.tif]

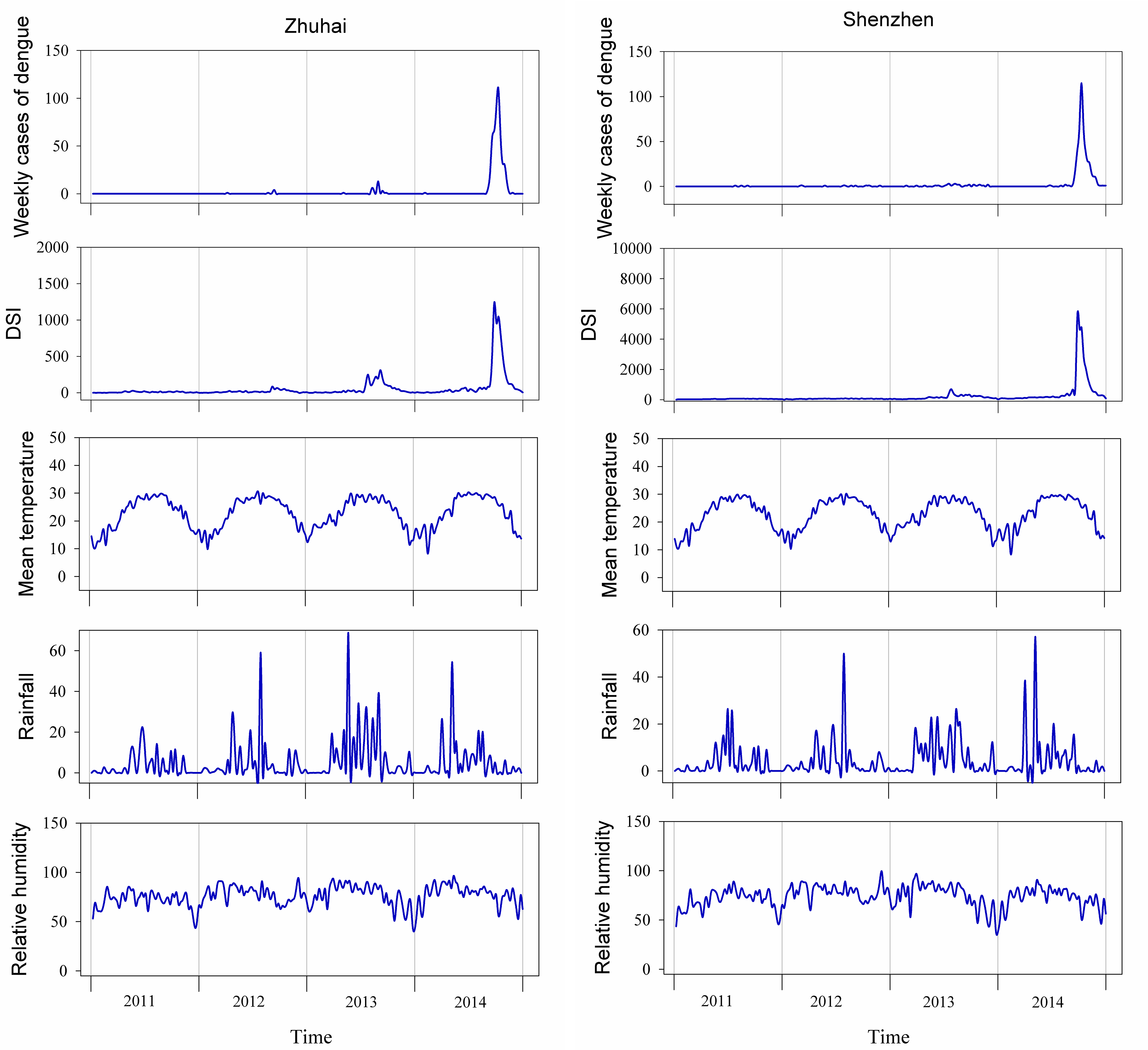

Supplement: S5 Fig — (TIF) [file pntd.0005973.s006.tif]

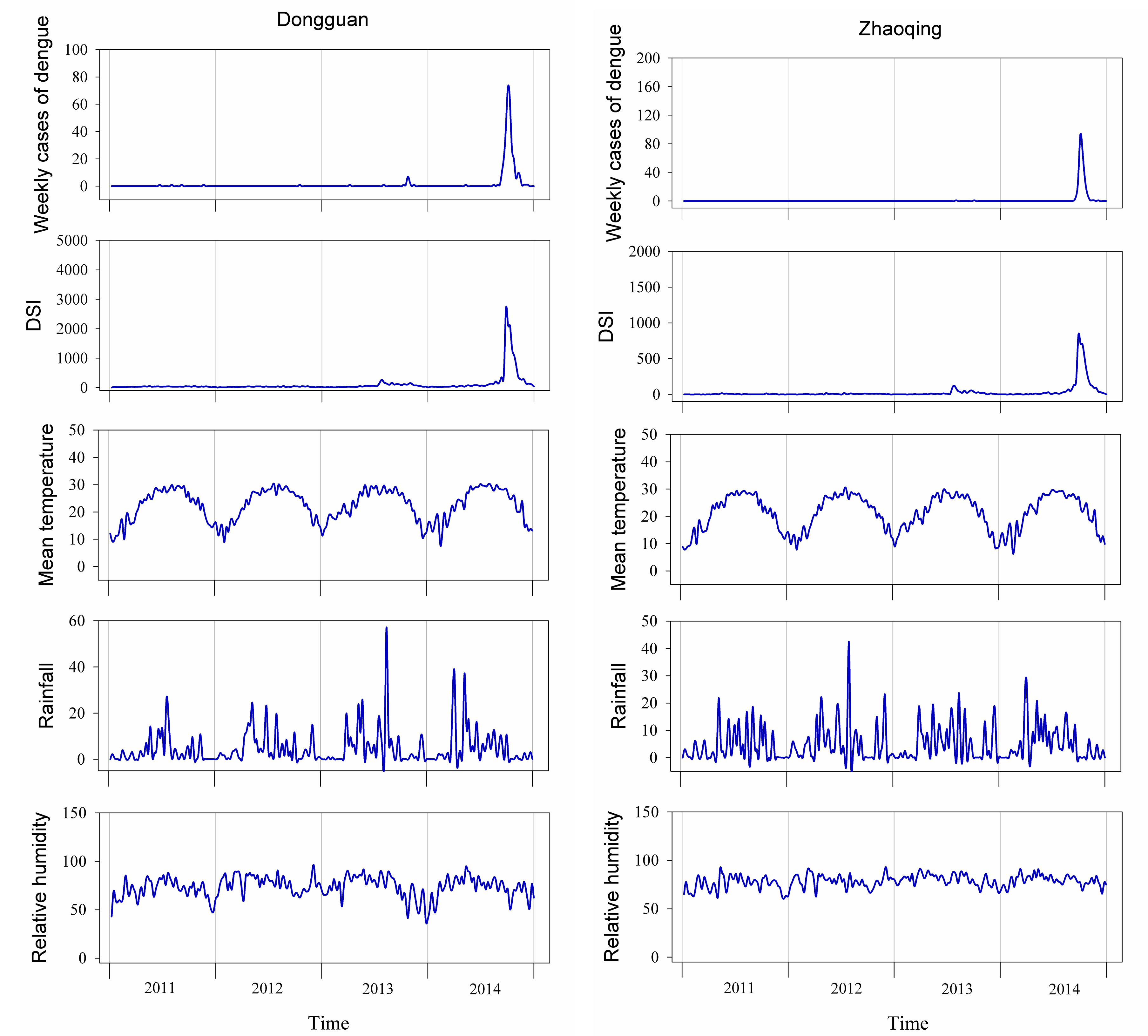

Supplement: S6 Fig — (TIF) [file pntd.0005973.s007.tif]

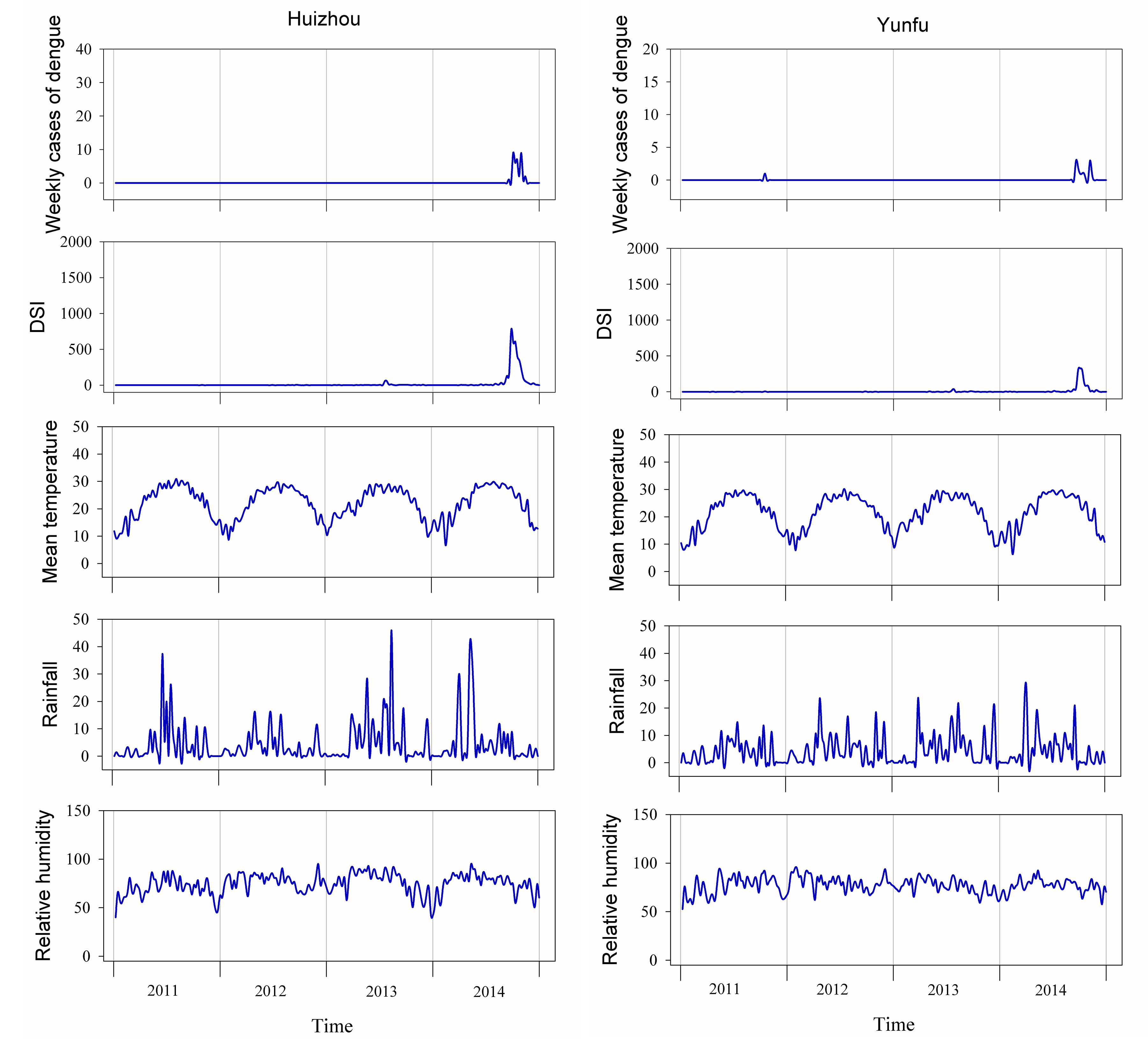

Supplement: S7 Fig — (TIF) [file pntd.0005973.s008.tif]

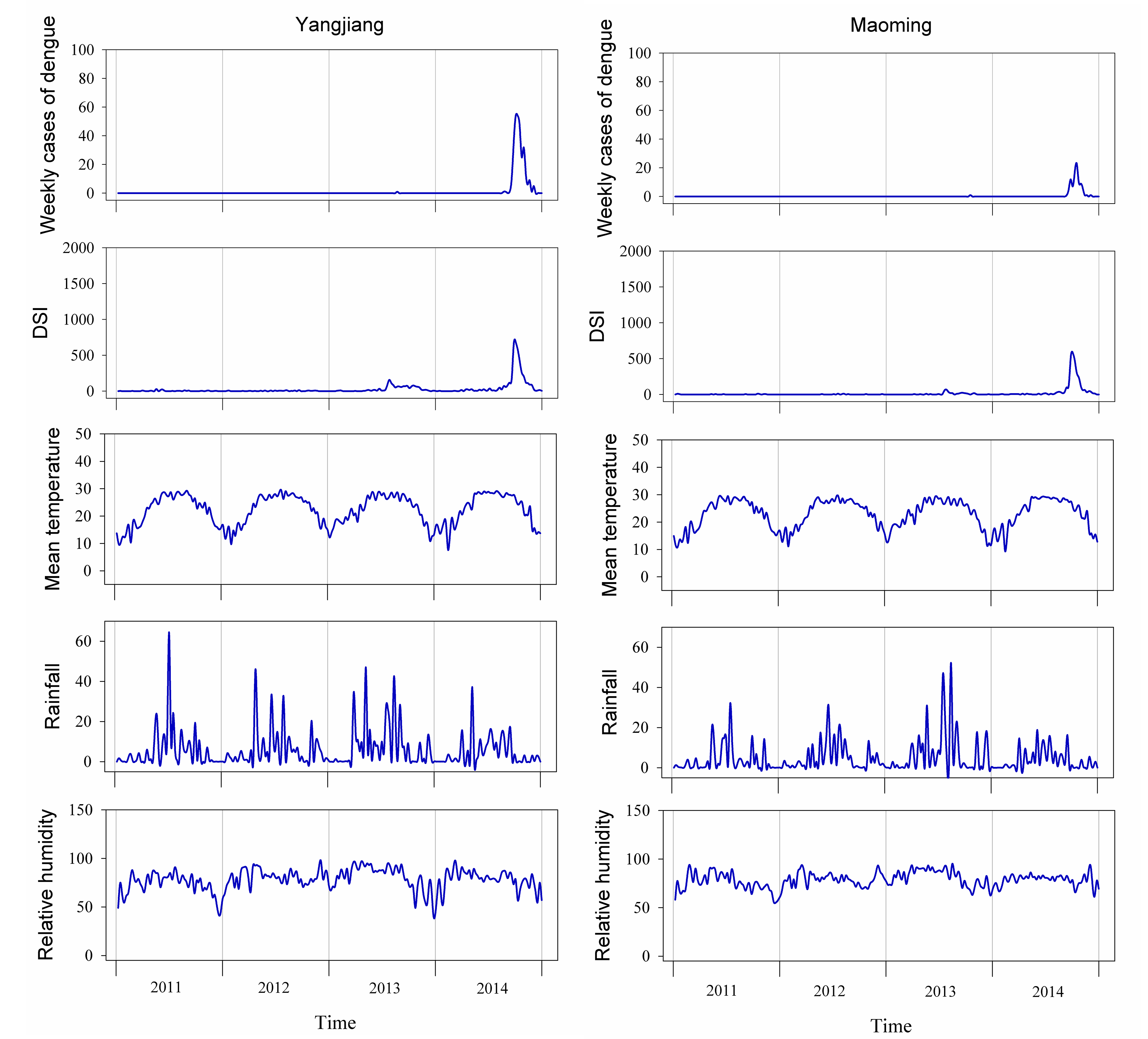

Supplement: S8 Fig — (TIF) [file pntd.0005973.s009.tif]

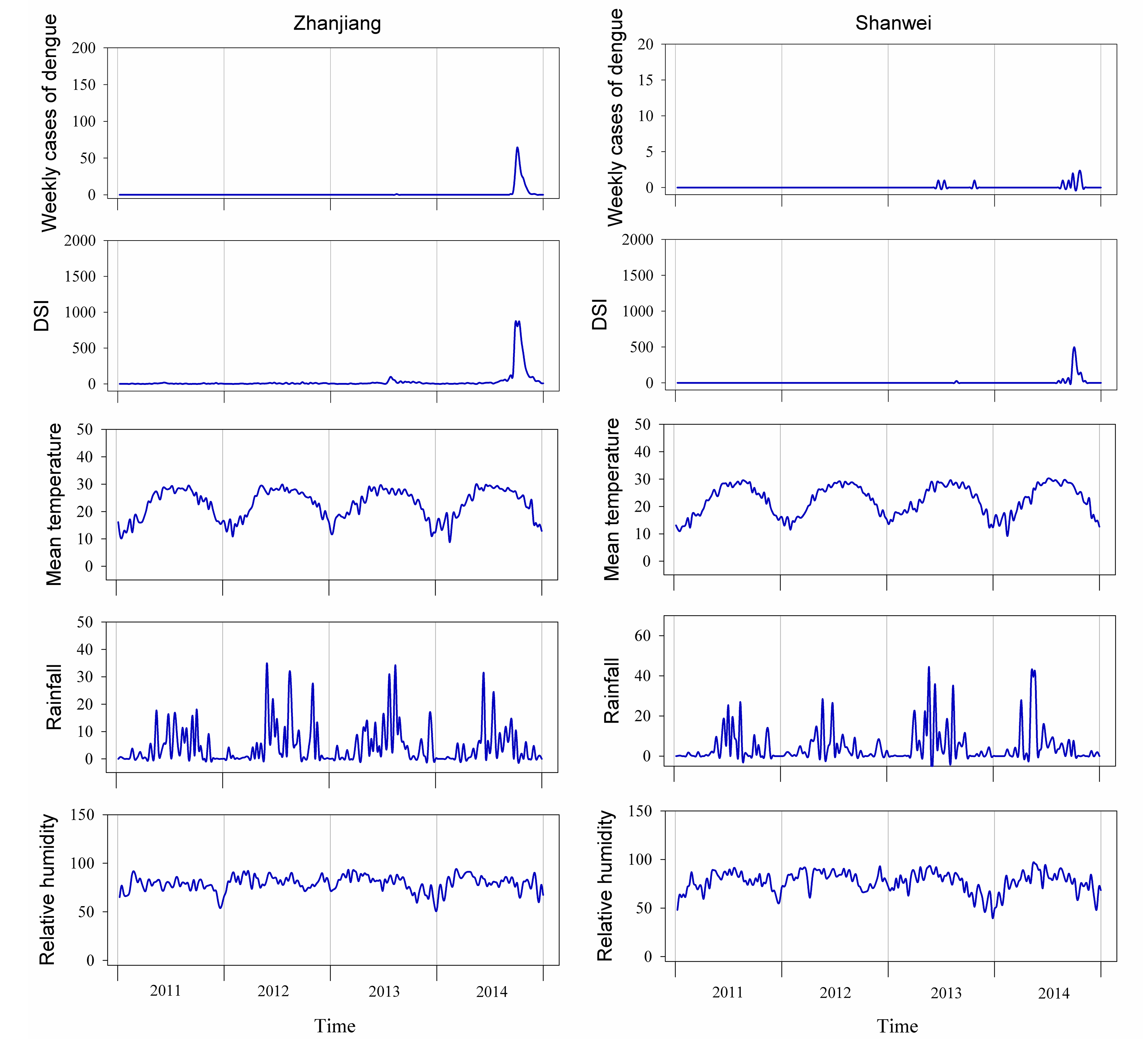

Supplement: S9 Fig — (TIF) [file pntd.0005973.s010.tif]

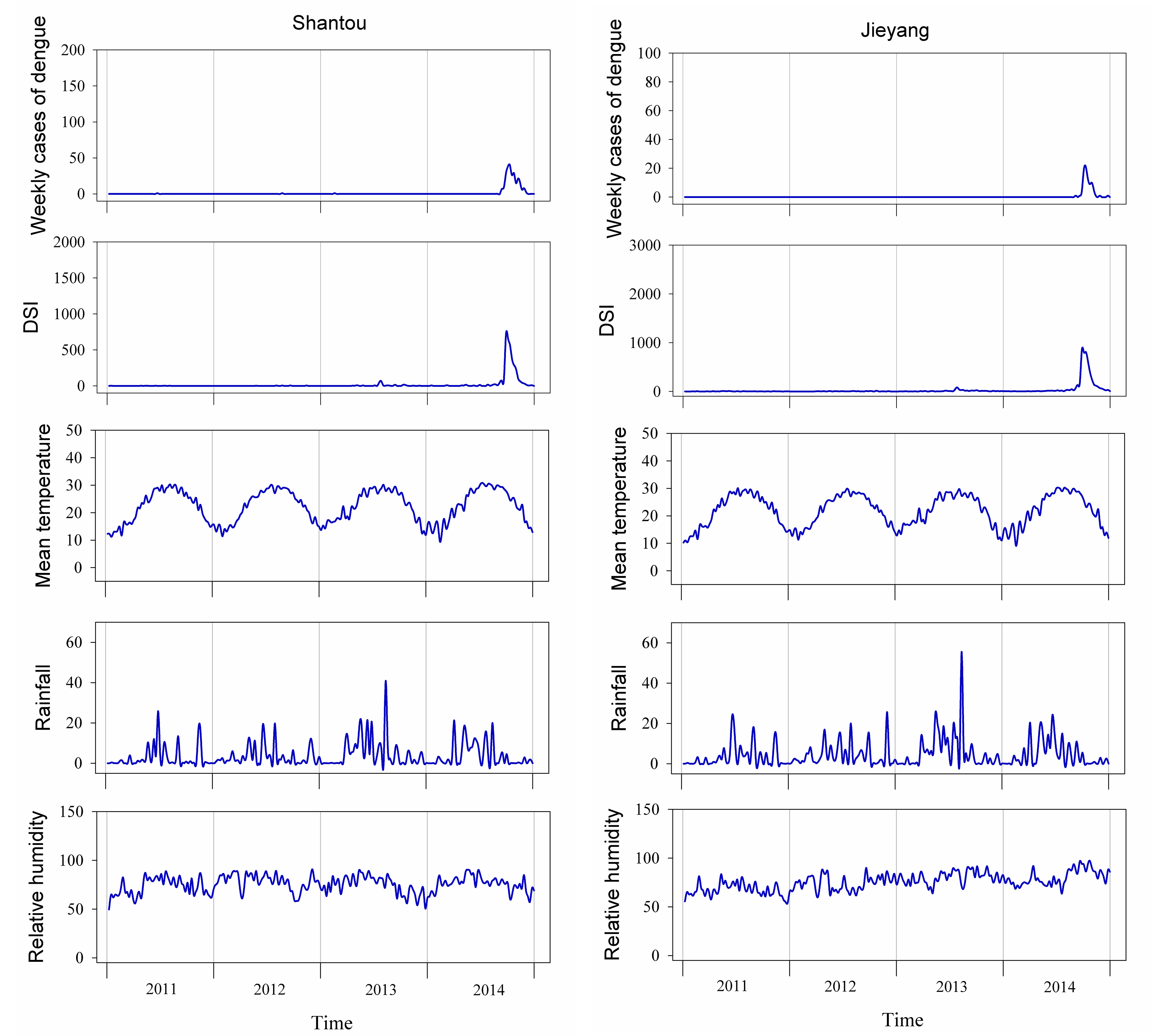

Supplement: S10 Fig — (TIF) [file pntd.0005973.s011.tif]

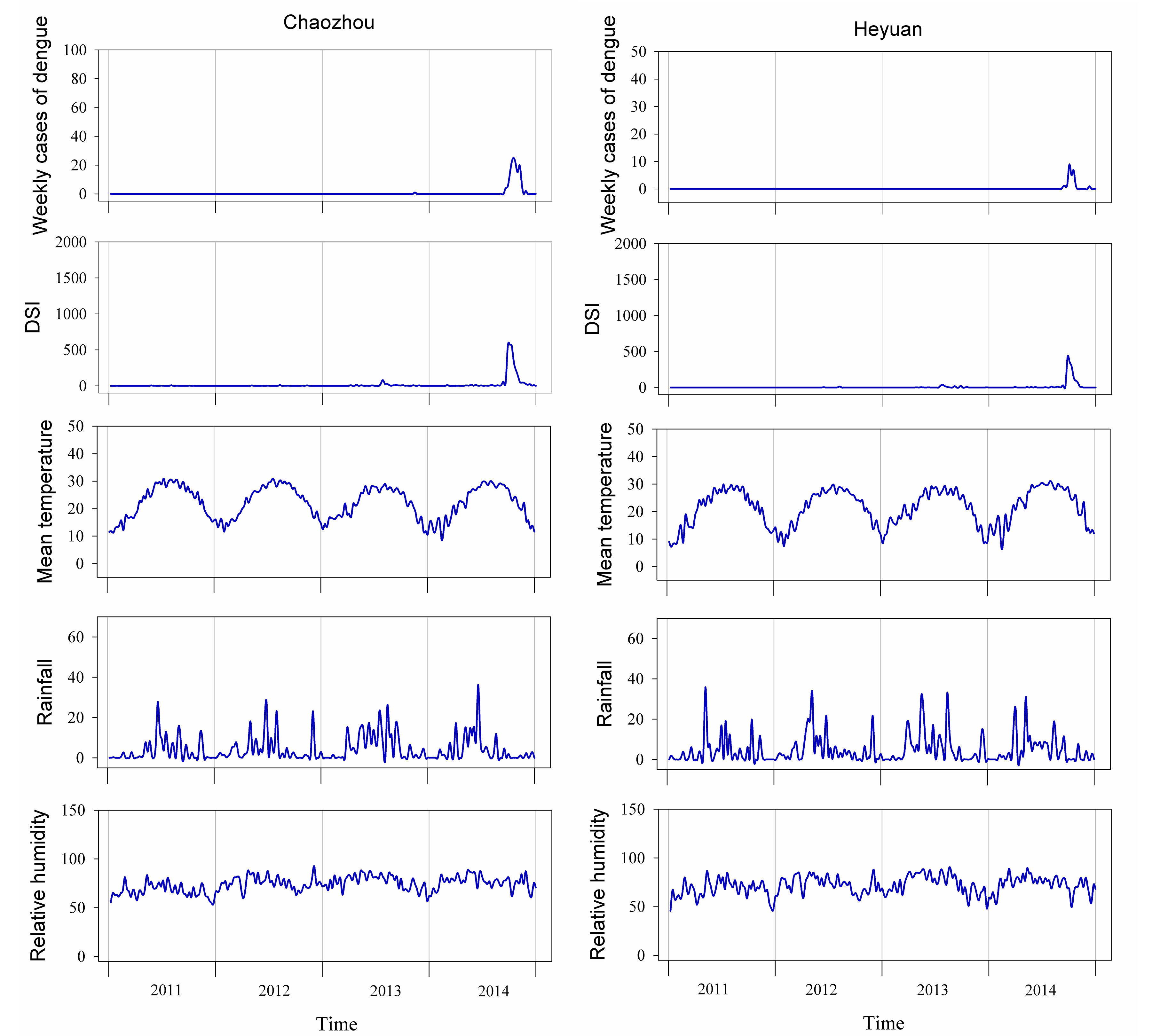

Supplement: S11 Fig — (TIF) [file pntd.0005973.s012.tif]

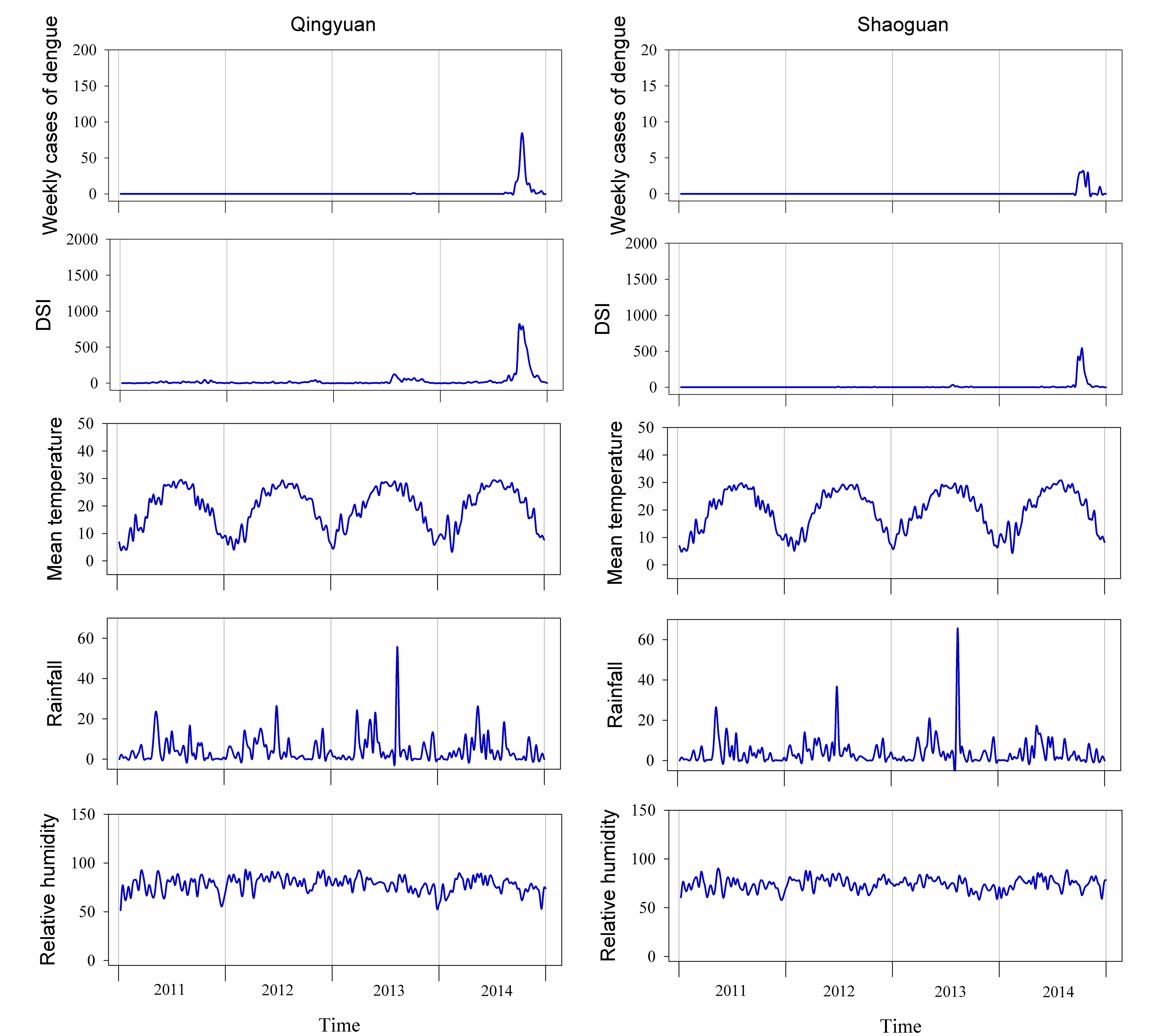

Supplement: S12 Fig — (TIF) [file pntd.0005973.s013.tif]

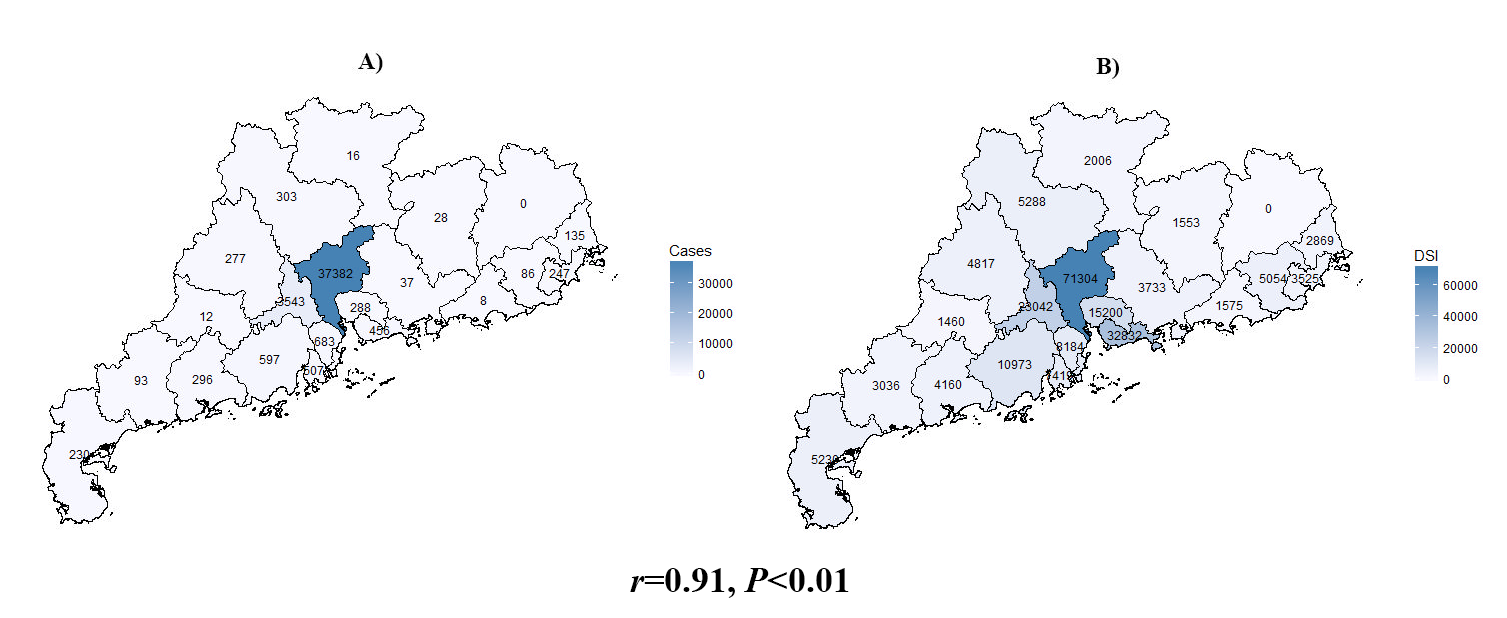

Supplement: S13 Fig — (A) Geographical distribution of dengue cases of 2014 in Guangdong. (B) Geographical distribution of DSI of 2014 in Guangdong. There was a significant correlation (Spearman correlation coefficient r = 0.91) between the geographical distribution of dengue incidence and that of DSI in Guangdong, China. (TIF) [file pntd.0005973.s014.tif]

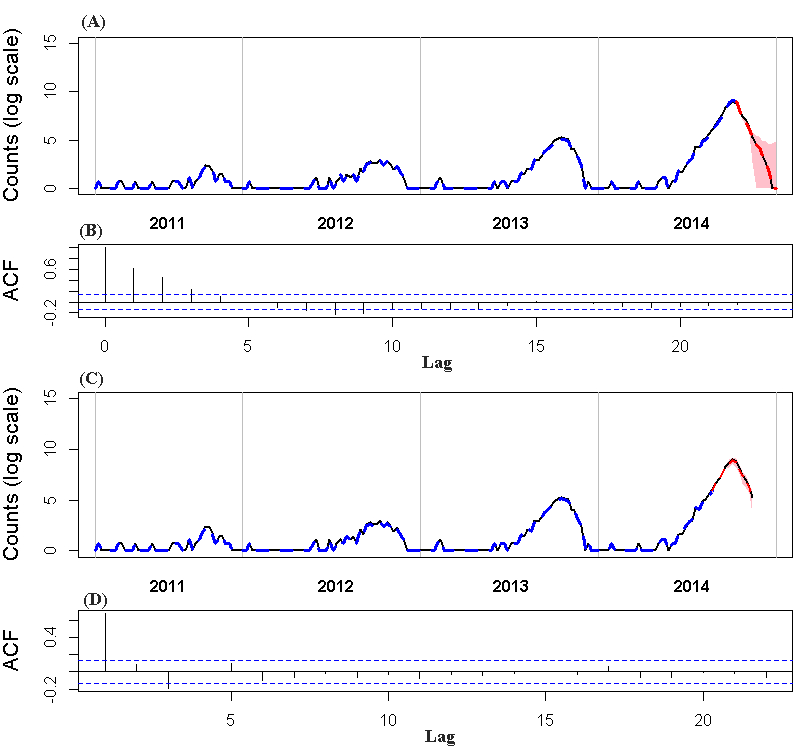

Supplement: S14 Fig — (A) Model forecasts using the SVR algorithm for the dengue epidemic period between the 41st to 53rd weeks (the last 12 weeks) in 2014. The black lines represent observed values, the blue dash lines denote model-based fitted values, the red dash lines correspond to model-based predicted values, and the pink contours represent the corresponding 95% prediction intervals. The observations and predictions of dengue case counts were expressed as a log-scale. (B) Residuals of the SVR model for the last 12 weeks forecasts were assessed using the autocorrelation function (ACF) plot. (C) Model forecasts using the SVR algorithm for the period between the 35th to 46th weeks which covers the outbreak in dengue incidence in 2014. (D) Residuals of the SVR model for the outbreak period forecasts were assessed using the ACF plot. (TIF) [file pntd.0005973.s015.tif]

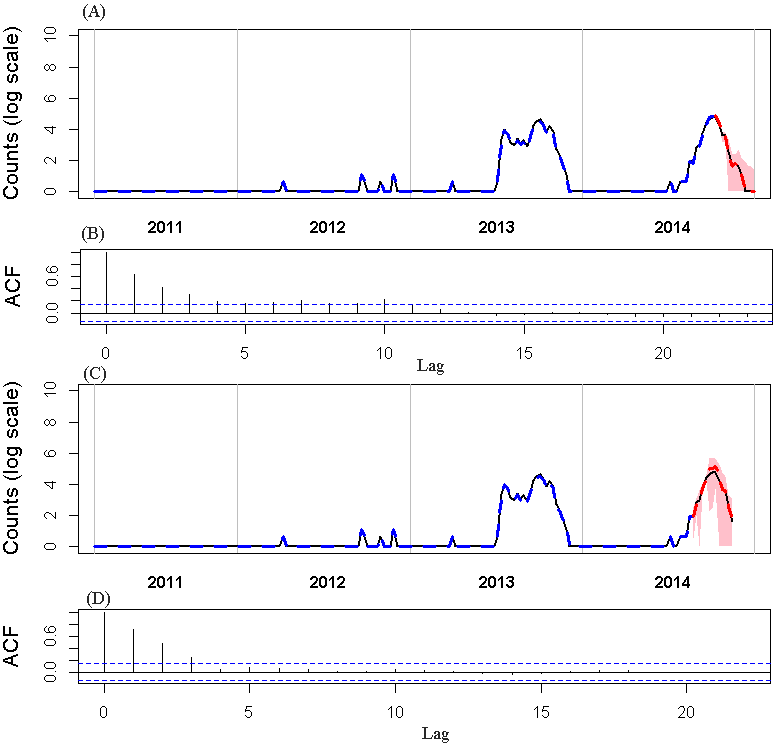

Supplement: S15 Fig — (A) Model forecasts using the SVR algorithm for the dengue epidemic period between the 41st to 53rd weeks (the last 12 weeks) in 2014. The black lines represent observed values, the blue dash lines denote model-based fitted values, the red dash lines correspond to model-based predicted values, and the pink contours represent the corresponding 95% prediction intervals. The observations and predictions of dengue case counts were expressed as a log-scale. (B) Residuals of the SVR model for the last 12 weeks forecasts were assessed using the autocorrelation function (ACF) plot. (C) Model forecasts using the SVR algorithm for the period between the 35th to 46th weeks which covers the outbreak in dengue incidence in 2014. (D) Residuals of the SVR model for the outbreak period forecasts were assessed using the ACF plot. (TIF) [file pntd.0005973.s016.tif]

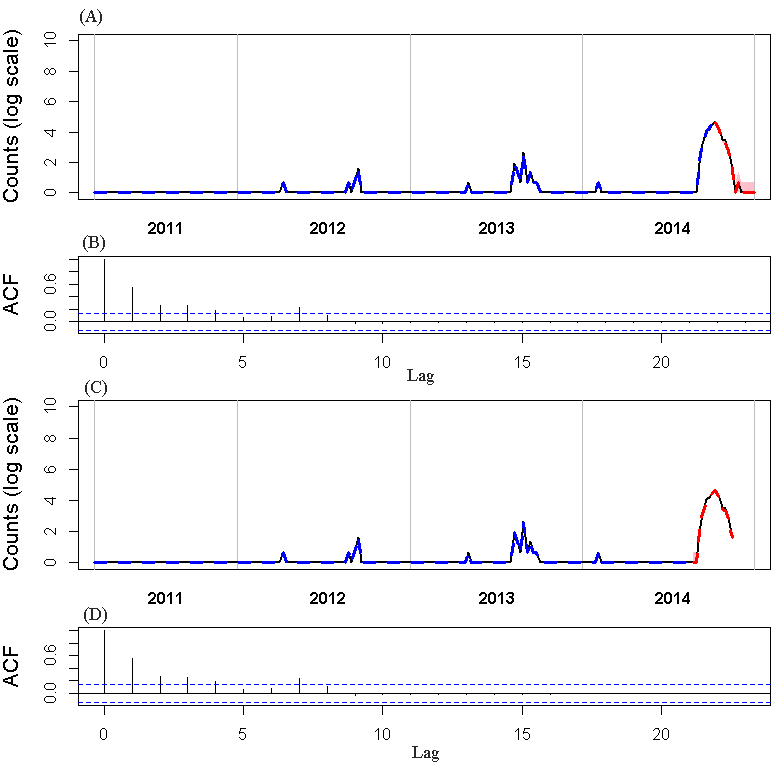

Supplement: S16 Fig — (A) Model forecasts using the SVR algorithm for the dengue epidemic period between the 41st to 53rd weeks (the last 12 weeks) in 2014. The black lines represent observed values, the blue dash lines denote model-based fitted values, the red dash lines correspond to model-based predicted values, and the pink contours represent the corresponding 95% prediction intervals. The observations and predictions of dengue case counts were expressed as a log-scale. (B) Residuals of the SVR model for the last 12 weeks forecasts were assessed using the autocorrelation function (ACF) plot. (C) Model forecasts using the SVR algorithm for the period between the 35th to 46th weeks which covers the outbreak in dengue incidence in 2014. (D) Residuals of the SVR model for the outbreak period forecasts were assessed using the ACF plot. (TIF) [file pntd.0005973.s017.tif]

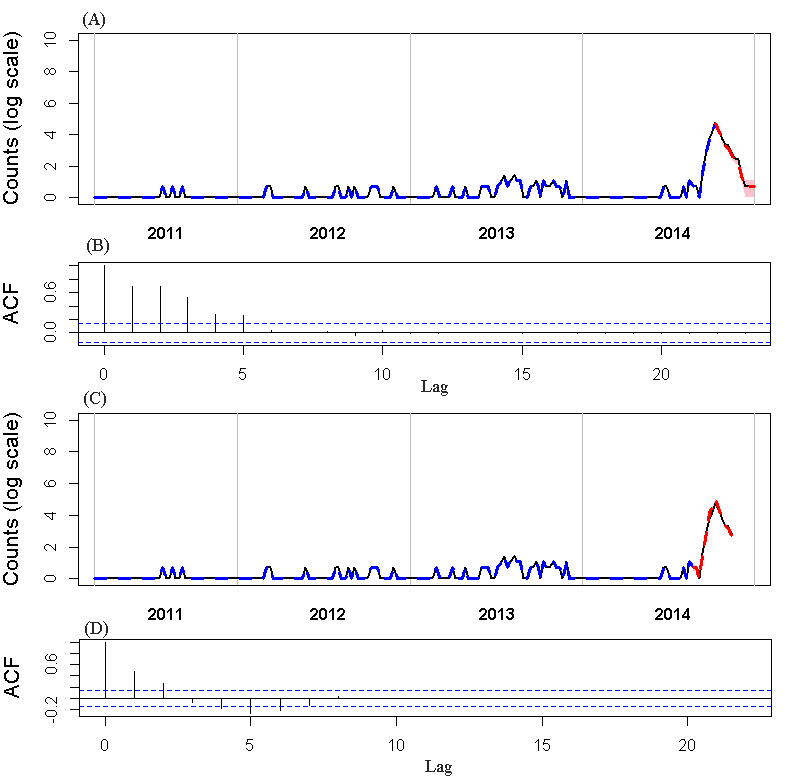

Supplement: S17 Fig — (A) Model forecasts using the SVR algorithm for the dengue epidemic period between the 41st to 53rd weeks (the last 12 weeks) in 2014. The black lines represent observed values, the blue dash lines denote model-based fitted values, the red dash lines correspond to model-based predicted values, and the pink contours represent the corresponding 95% prediction intervals. The observations and predictions of dengue case counts were expressed as a log-scale. (B) Residuals of the SVR model for the last 12 weeks forecasts were assessed using the autocorrelation function (ACF) plot. (C) Model forecasts using the SVR algorithm for the period between the 35th to 46th weeks which covers the outbreak in dengue incidence in 2014. (D) Residuals of the SVR model for the outbreak period forecasts were assessed using the ACF plot. (TIF) [file pntd.0005973.s018.tif]

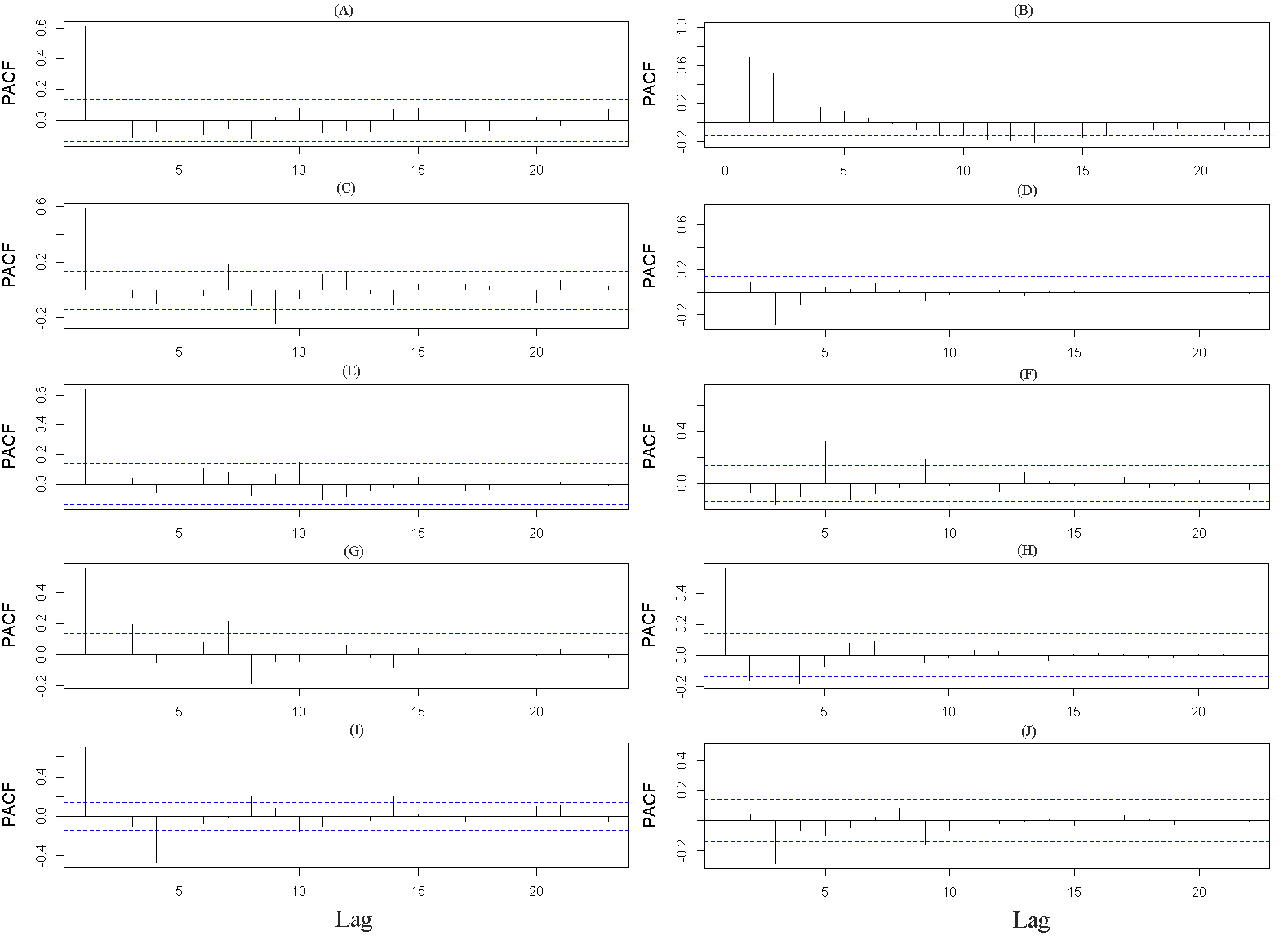

Supplement: S18 Fig — (A) PACF analysis for the SVR model forecasting the period between the 41st to 53rd weeks of 2014 in Guangzhou. (B) PACF analysis for the SVR model forecasting the period between the 35th to 46th weeks of 2014 in Guangzhou. (C) PACF analysis for the SVR model forecasting the period between the 41st to 53rd weeks of 2014 in Foshan. (D) PACF analysis for the SVR model forecasting the period between the 35th to 46th weeks of 2014 in Foshan. (E) PACF analysis for the SVR model forecasting the period between the 41st to 53rd weeks of 2014 in Zhongshan. (F) PACF analysis for the SVR model forecasting the period between the 35th to 46th weeks of 2014 in Zhongshan. (G) PACF analysis for the SVR model forecasting the period between the 41st to 53rd weeks of 2014 in Zhuhai. (H) PACF analysis for the SVR model forecasting the period between the 35th to 46th weeks of 2014 in Zhuhai. (I) PACF analysis for the SVR model forecasting the period between the 41st to 53rd weeks of 2014 in Shenzhen. (J) PACF analysis for the SVR model forecasting the period between the 35th to 46th weeks of 2014 in Shenzhen. (TIF) [file pntd.0005973.s019.tif]

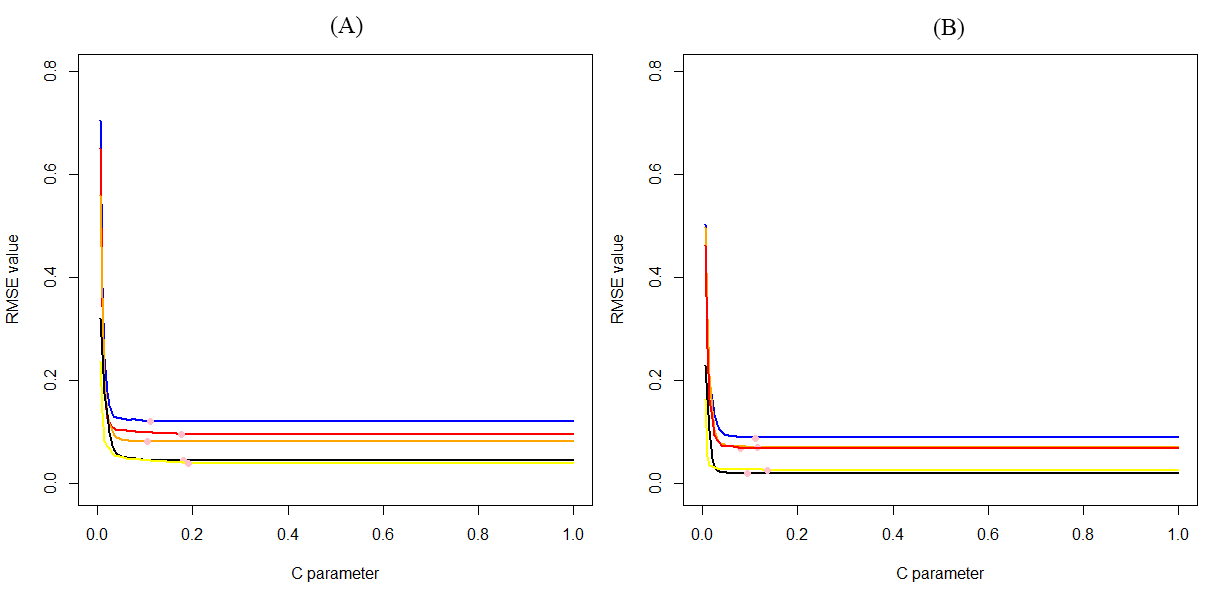

Supplement: S19 Fig — (A) Model forecasts for the dengue epidemic period between the 41st to 53rd weeks (the last 12 weeks) in 2014. (B) Model forecasts for the period between the 35th to 46th weeks which covers the outbreak in dengue incidence in 2014. A cross-validation approach with root-mean-square error (RMSE) as an indicator of model was performance to select an optimal SVR model. Several SVR models were trained for different values of the C parameter, and the most superior one corresponding to the lowest RMSE value was identified. Blue: Guangzhou. Red: Foshan. Orange: Zhongshan. Black: Zhuhai. Yellow: Shenzhen. The optimal values of RMSE for the SVR models are denoted using the pink solid dots. (TIF) [file pntd.0005973.s020.tif]

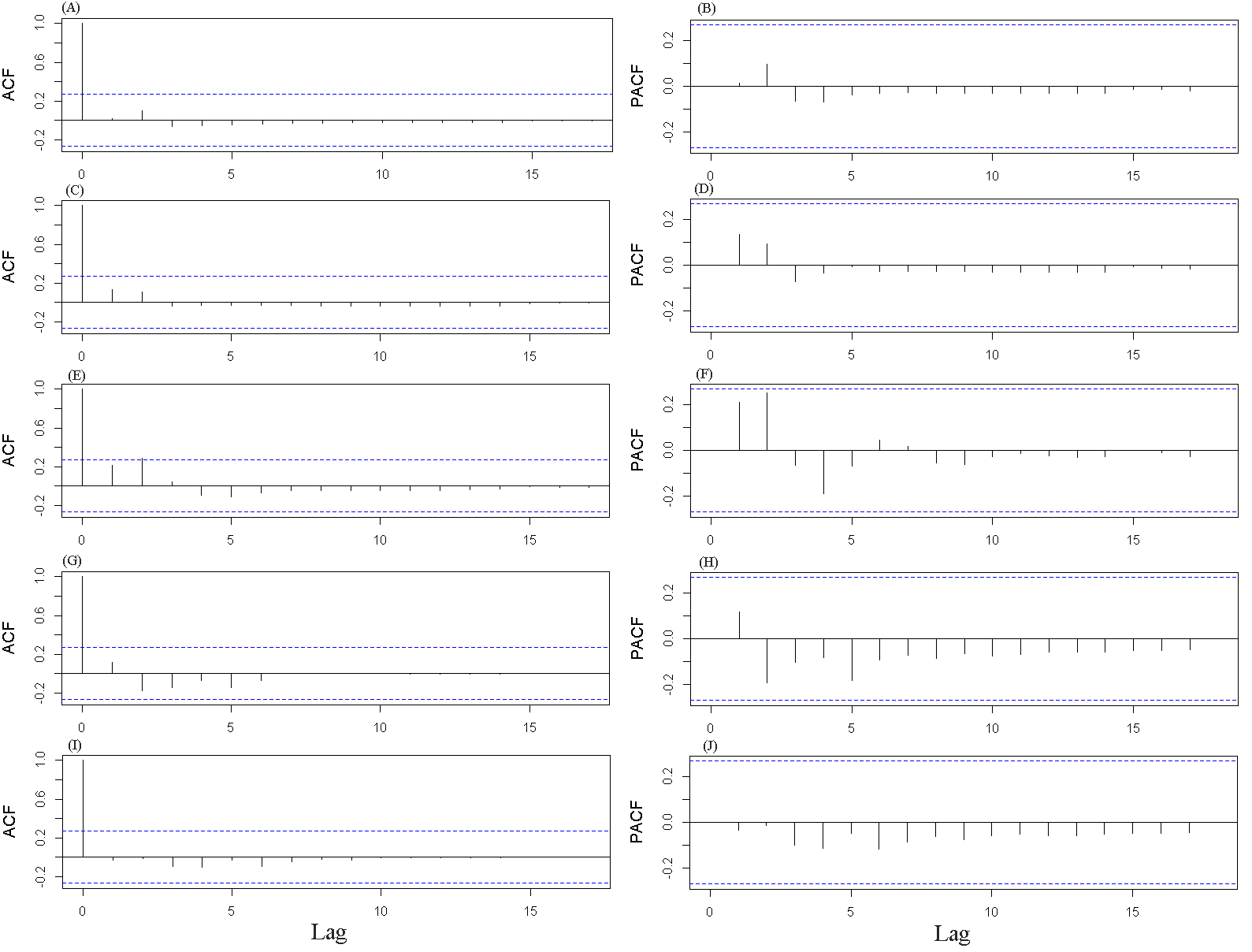

Supplement: S20 Fig — (A) ACF analysis of the 1-week-ahead predictions in Guangzhou. (B) PACF analysis of the 1-week-ahead predictions in Guangzhou. (C) ACF analysis of the 1-week-ahead predictions in Foshan. (D) PACF analysis of the 1-week-ahead predictions in Foshan. (E) ACF analysis of the 1-week-ahead predictions in Zhongshan. (F) PACF analysis of the 1-week-ahead predictions in Zhongshan. (G) ACF analysis of the 1-week-ahead predictions in Zhuhai. (H) PACF analysis of the 1-week-ahead predictions in Zhuhai. (I) ACF analysis of the 1-week-ahead predictions in Shenzhen. (J) PACF analysis of the 1-week-ahead predictions in Shenzhen. (TIF) [file pntd.0005973.s021.tif]
