# Supplementary material for: Developing a dengue forecast model using machine learning: A case study in China
Source: PLoS Negl Trop Dis. 2017 Oct 16;11(10):e0005973. doi: 10.1371/journal.pntd.0005973 (PMC5658193; doi:10.1371/journal.pntd.0005973)
Supplement: S1 Table — (DOCX) [file pntd.0005973.s022.docx]

**S1 Table. Search keywords from Baidu Index website used in this study.** The primary search terms were in bold. The keywords in italics were the related keywords derived from the primary search terms.

| Variable | Chinese name | English name | Variable | Chinese name | English name |
| --- | --- | --- | --- | --- | --- |
| X1 | **登革热** | **dengue fever** | X27 | *37人染登革热* | *37 people infected with dengue fever* |
| X2 | **登革热病** | **dengue fever sickness** | X28 | *登革热病是由哪种动物传染的* | *what kind of animal transmits the disease of dengue fever* |
| X3 | **登革热病毒** | **dengue fever virus** | X29 | *登革热传染途径* | *infection routes of dengue fever* |
| X4 | **登革热病例** | **dengue case** | X30 | *登革热概念股* | *dengue fever concept stocks* |
| X5 | **登革热症状** | **dengue symptom** | X31 | *登革热及登革出血热* | *dengue fever and dengue hemorrhagic fever* |
| X6 | **发烧** | **fever** | X32 | *登革热检测试剂盒* | *dengue fever assay kit* |
| X7 | **皮疹** | **rash** | X33 | *登革热是什么病* | *what is the disease of dengue fever* |
| X8 | **肌肉痛** | **muscle pain** | X34 | *登革热预防知识* | *knowledge of dengue fever prevention* |
| X9 | **骨痛** | **bone pain** | X35 | *登革热诊断标准* | *diagnostic criteria for dengue fever* |
| X10 | **关节疼痛** | **joint pain** | X36 | *广东1145人患登革热* | *1145 people getting dengue fever in Guangdong* |
| X11 | **伊蚊** | **aedes** | X37 | *广东登革热病例破2万* | *dengue cases more than 20 thousands in Guangdong* |
| X12 | **蚊子** | **mosquito** | X38 | *台湾 登革热* | *Taiwan dengue fever* |
| X13 | *登革热治疗* | *treatment for dengue fever* | X39 | *台湾登革热最新疫情* | *the latest epidemic of dengue fever in Taiwan* |
| X14 | *预防登革热* | *prevent dengue fever* | X40 | *白纹伊蚊* | *aedes albopictus* |
| X15 | *登革热的症状* | *symptoms of dengue fever* | X41 | *伊蚊属* | *genus aedes* |
| X16 | *登革热潜伏期* | *latent period of dengue fever* | X42 | *花蚊子* | *striped mosquito* |
| X17 | *登革热疫苗* | *dengue vaccines* | X43 | *毒蚊子* | *poisonous mosquito* |
| X18 | *男婴感染登革热* | *baby boy infected with dengue fever* | X44 | *蚊子咬了怎么消肿止痒* | *how to relieve itching and swelling when be* *bitten by mosquitoes* |
| X19 | *广州登革热疫情* | *dengue epidemic in Guangzhou* | X45 | *防蚊子* | *anti-mosquito* |
| X20 | *登革热预防* | *dengue fever prevention* | X46 | *蚊子叮咬* | *mosquito bite* |
| X21 | *登革热疫情* | *dengue fever epidemic* | X47 | *蚊子咬了怎么办* | *how to do when be bitten by mosquitoes* |
| X22 | *如何预防登革热* | *how to prevent dengue fever* | X48 | *驱蚊子* | *drive the mosquitoes* |
| X23 | *登革热图片* | *dengue fever pictures* | X49 | *杀蚊子* | *kill the mosquitoes* |
| X24 | *中山登革热* | *Zhongshan dengue fever* | X50 | *灭蚊子* | *destroy the mosquitoes* |
| X25 | *后背疼痛是什么原因* | *what’s the cause of back pain* | X51 | *被毒蚊子咬了怎么办* | *how to do when be bitten by poisonous mosquitoes* |
| X26 | *蚊香* | *mosquito coil* |  |  |  |
